# Changes in Soybean (*Glycine max* L.) Flour Fatty-Acid Content Based on Storage Temperature and Duration

**DOI:** 10.3390/molecules23102713

**Published:** 2018-10-21

**Authors:** Mayakrishnan Prabakaran, Kyoung-Jin Lee, Yeonju An, Chang Kwon, Soyeon Kim, Yujin Yang, Ateeque Ahmad, Seung-Hyun Kim, Ill-Min Chung

**Affiliations:** 1 Department of Crop Science, College of Sanghuh Life Science, Konkuk University, 120 Neungdong-ro, Gwangjin-gu, Seoul 05029, Korea; prabakarannitt@gmail.com (M.P.); jin331@konkuk.ac.kr (K.-J.L.); ayj3043@konkuk.ac.kr (Y.A.); chang794@konkuk.ac.kr (C.K.); hellosy1@konkuk.ac.kr (S.K.); jin9031@konkuk.ac.kr (Y.Y.); kshkim@konkuk.ac.kr (S.-H.K.); 2 Process Chemistry and Technology Department, CSIR-Central Institute of Medicinal and Aromatic Plants, Lucknow 226015, India; ateeque97@gmail.com

**Keywords:** soybean, fatty acids, stability, gas chromatography/flame ionization detector (GC–FID), autoxidation

## Abstract

Soybeans are low in saturated fat and a rich source of protein, dietary fiber, and isoflavone; however, their nutritional shelf life is yet to be established. This study evaluated the change in the stability and quality of fatty acids in raw and roasted soybean flour under different storage temperatures and durations. In both types of soybean flour, the fatty-acid content was the highest in the order of linoleic acid (18-carbon chain with two double bonds; C18:2), oleic acid (C18:1), palmitic acid (C16:0), linolenic acid (18:3), and stearic acid (C18:0), which represented 47%, 26%, 12%, 9%, and 4% of the total fatty-acid content, respectively. The major unsaturated fatty acids of raw soybean flour—oleic acid, linoleic acid, and linolenic acid—decreased by 30.0%, 94.4%, and 97.7%, and 38.0%, 94.8%, and 98.0% when stored in polyethylene and polypropylene film, respectively, after 48 weeks of storage under high-temperature conditions. These values were later increased due to hydrolysis. This study presents the changes in composition and content of two soybean flour types and the changes in quality and stability of fatty acids in response to storage temperature and duration. This study shows the influence of storage conditions and temperature on the nutritional quality which is least affected by packing material.

## 1. Introduction

Soybean plants are presumed to be native to northeastern China and were not widely distributed, except in China and Japan, until 1890, even after their introduction in Europe. Soybean can easily be mass produced, and the soybean plant is considered to be one of the principal plants that can solve world hunger [1]. Soybean contains low amounts of saturated fat and is a great source of high-quality protein, dietary fiber, and isoflavone, which makes it unique among other legumes [2].

Soybean oil and protein received great commercial interest before the recent attention on soybean protein. According to a United States Department of Agriculture (USDA) report, soybean oil is the second largest vegetable oil produced worldwide after palm oil. Most beans are very low in fat, but soybeans have an exceptionally high fat content [3]. Soybean fat has a uniquely high content of polyunsaturated fatty acids, such as linoleic (18-carbon chain with two double bonds; C18:2) and linolenic (C18:3) acids. They also contain a substantial amount of monounsaturated fatty acids, such as oleic acid (C18:1), and moderate amounts of saturated fatty acids, such as palmitic (C16:0) and stearic acids (C18:0). The predominant fatty acid is linoleic acid, which accounts for ~53% of the total fatty-acid content of soybeans. It is also notable that linolenic-acid content is approximately only 7–8% [3] in other beans, and on account of this low fat content, the dietary contribution of beans to α-linolenic acid is minor except for soybeans. Full-fat soybeans with high fat content can contribute significantly to the intake of α-linolenic acid which is considered an essential fatty acid for human nutrition [4]. The n-3 fatty acids such as α-linolenic acid, docosahexaenoic acid (DHA), and eicosapentaenoic acid (EPA) inhibit the synthesis of triglycerides and very-low-density lipoproteins (VLDLs) in the liver, which were shown to have a major effect in lowering triglyceride levels in the blood [5].

Traditionally, Koreans consume large quantities of soybeans and soy-containing foods to overcome the risk of protein deficiency caused by a predominantly rice-based diet, which may be responsible for the low incidence of hormone-related diseases, such as breast and prostate cancer [6]. Global soybean consumption steadily increased at an average annual rate of 4%, and the annual soybean consumption in Korea increased from 4.4 kg per capita in 1965 to 8.5 kg per capita in 2003 [7]. The main non-fermented soybean foods consumed in Korea are soy milk, tofu, and bean sprouts. Fermented foods include soy sauce, miso, and chungkukjang [8].

Soybean flour is produced by grinding raw or roasted soybeans to a fine particle size of 100 mesh or less [9]. Soybean flour can be found in full-fat or defatted types. Soybean flour is used in a variety of food products, such as cereals, animal milk replacers, bakery mixes, or beverages, and can be fully, medium, or lightly cooked. Uncooked flour is also used as a bleaching agent in white bread [10] and is widely used to raise the protein content in foods, such as bread and pasta [11].

One common problem of soybean products is their beany taste due to the lipoxygenase-catalyzed oxidation of unsaturated fatty acids in soybean oil to volatile compounds [12]. Heat treatment is the most common method used to inactivate lipoxygenase in soybeans, which leads to cooked and toasted flavors. Heat treatment also inactivates anti-nutritional factors, such as the trypsin inhibitor that inhibits digestion and enhances palatability by softening the tissue [13]. The contained nutrients may be destroyed if soybeans are over-heated. Therefore, proper heat treatment is the most important requirement for the maximum retention of the essential nutrients in soybean flour [14].

Because lipid oxidation produces low-molecular off-flavor compounds and leads to the loss of essential fatty acids, it is a very important reaction that determines the nutritional, functional, and sensory characteristics of foods, as well as their storage stability. During the initial stages of the oxidation process, hydroperoxides accumulate as primary oxidation products, which thereafter decompose to secondary oxidation products, such as alcohols, aldehydes, free fatty acids, and ketones, resulting in rancidity [15]. The storage stability of soybean flour is related to its unsaturated fat content; in particular, linolenic acid is the main cause of flavor reversion [16].

The main roles of food packaging are to contain and protect food from external influences and damages, and to provide ingredient and nutritional information to consumers. Food packaging can extend the shelf life and maintain or enhance the food quality and consistency. Polyethylene and polypropylene are the most widely used food packaging materials due to their suitable properties, such as flexibility, strength, stability, lightness, chemical resistance, and moisture prevention, and because these materials can easily be processed and, therefore, reused and recycled. Polyethylene is the simplest and most inexpensive plastic. There are two basic categories of polyethylene: high and low density. Low-density polyethylene is especially flexible, strong, tough, and resistant to moisture. Since low-density polyethylene is relatively transparent, it is often used when film application and heat sealing are required. Polypropylene is denser, harder, and more transparent than polyethylene, and has a high resistance to chemicals and moisture. Polypropylene also has a high melting point (160 °C), which makes it suitable for applications where thermal resistance is required [17].

Studies in various parts of the world assessed the amino-acid composition, protein fractionation, and electrophoretic characteristics of protein, which is the main component of soybeans [18,19,20]. Studies were also conducted on the lipid component, in addition to it assessment related to the processing and storage of soybeans [21,22]. Changes in physical characteristics and nutritional composition were also identified in genotypically different soybean (*Glycine max* (L.) Merrill) varieties [23].

Though many studies explaining the composition of soybean flour were carried out, very few studies were done to identify the exact storage conditions to enhance the shelf life of soybean flour during storage. Lipid oxidation is a major cause in affecting the nutritional quality of soybean during storage. Lipid oxidation is well known for the development of off-flavors and loss of nutrients leading to the formation of potentially toxic compounds ending in the deterioration of food. Lipids are susceptible to spontaneous oxidation (i.e., autoxidation) and oxidation in the presence of catalytic systems, such as heat, light, and enzymes, leading to complex processes of thermal oxidation, photooxidation, and enzymatic oxidation, respectively [24]. It is generally accepted that lipid autoxidation is a mechanism (a simplified schematic explanation of the mechanism of autoxidation is given in Supplementary Materials Figure S1) that leads to a series of complex chemical changes through free-radical chain mechanisms that undergo three steps: initiation, propagation, and termination.

Thus, identifying the exact shelf life of soybean flour is crucial through identifying the proper storage conditions (i.e., temperature and humidity) and maximum storage duration of soybean flour. In this study, we aimed to evaluate storage stability and changes in soybean flour quality by monitoring changes in fatty-acid content of raw and roasted soybean flour under different storage conditions (i.e., temperature and duration). The effects of packing material and storage condition on the nutritional quality of soybean flour varieties were identified. This study will help in finding suitable conditions in order to retain the nutritional value of soybean flour even after storage. 

## 2. Results and Discussion

### 2.1. Lipid Oxidation

Lipid oxidation is a complex process in which the rate and process are affected by various factors, such as the fatty-acid profile, positional distribution of fatty acids, lipid class, minor component, and environmental factors. Lipids are not only susceptible to oxidative change during storage, but also during processing (roasting and deep frying) of the lipid-containing foods. The oxidation sensitivity of lipids depends primarily on the fatty-acid composition, specifically, their degree of unsaturation. Unsaturated fatty acids are the main reactants which undergo significant compositional changes during oxidation and are, therefore, an indirect measure for the extent of oxidation. The oxidation rates of stearic (C18:0), oleic (C18:1), linoleic (C18:2), and linolenic acids (C18:3) were reported to be in the ratio of 1:100:1200:2500 [25]. In this study, we measured the degree of oxidation in soybean flour under different storage conditions by evaluating the fatty-acid composition and content changes as indicators for storage stability.

### 2.2. Fatty Acid Composition and Content According to Storage Conditions

Before storage, the total fatty-acid contents of the raw soybean flour and the roasted soybean flour were 137.98 mg∙g^−1^ and 135.56 mg∙g^−1^, respectively. In both types of soybean flour, the fatty-acid content was the highest in the order of linoleic (C18:2), oleic (C18:1), palmitic (C16:0), linolenic (18:3), and stearic acids (C18:0), which represented 47%, 26%, 12%, 9%, and 4% of the total fatty-acid content, respectively. Our findings are consistent with those of previous studies that showed that linoleic (C18:2), oleic (C18:1), and palmitic acids (C16:0) were the major fatty acids with relative contents of more than 10% in soybean flour. The findings of this study differed slightly from those of previous studies that analyzed the fatty-acid composition of recommended soybean varieties in Korea; however, the differences are expected to be due to the difference in variety, which showed a similar tendency [22,26]. Previous studies showed that the protein content varies slightly, while the fat content varies greatly among soybean varieties [21]. 

The fatty-acid composition and content changes due to storage conditions are shown in Supplementary Materials Tables S1–S12 and Figures S2–S6.

**Figure 1:**
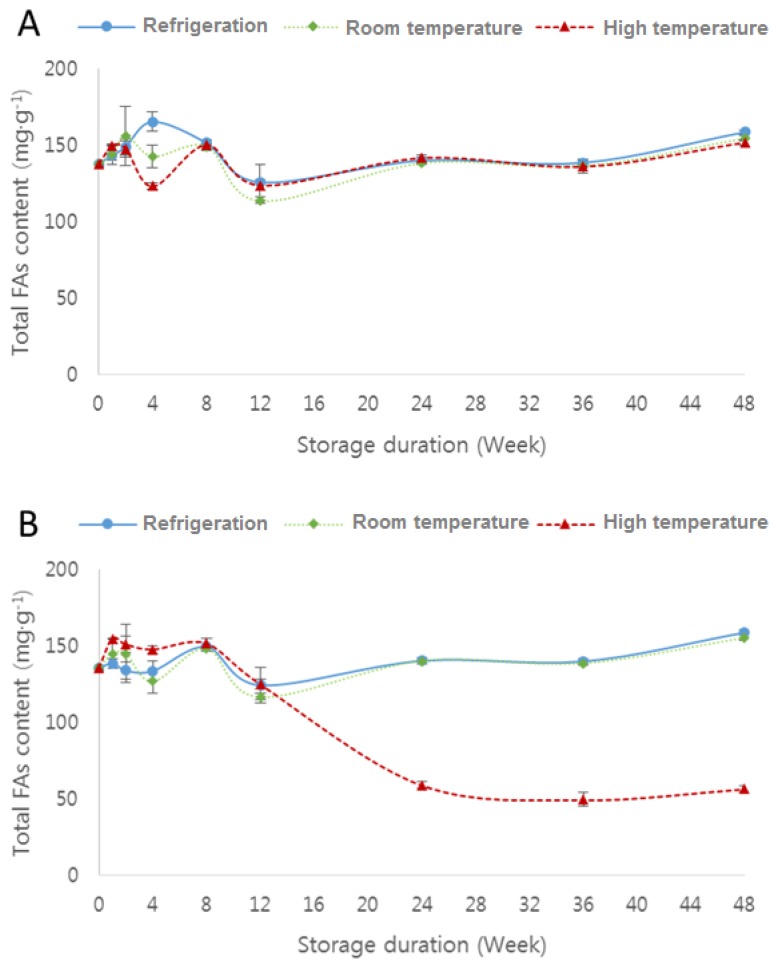
Changes in total fatty-acid (FA) content of raw soybean flour (**A**) and roasted soybean flour (**B**) packed in polyethylene film under different storage temperature conditions: refrigeration (4 °C), room temperature (20 °C), and high temperature (45 °C).

**Figure 2:**
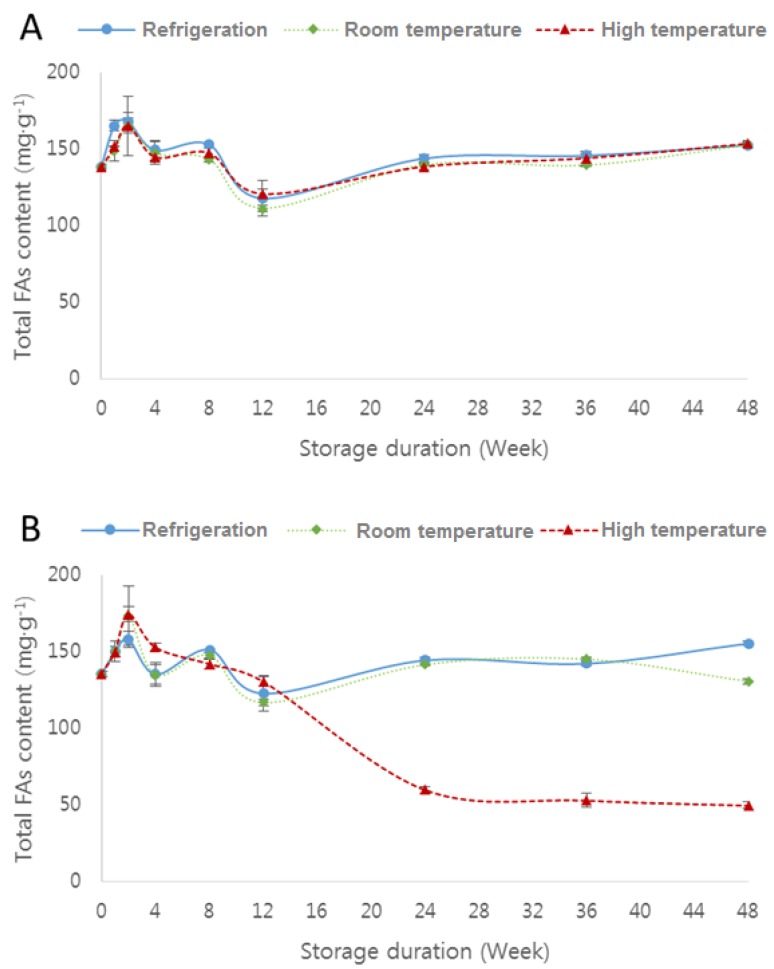
Changes in total fatty-acid (FA) content of raw soybean flour (**A**) and roasted soybean flour (**B**) packed in polypropylene film under different storage temperature conditions: refrigeration (4 °C), room temperature (20 °C), and high temperature (45 °C).

Figure S7 (Supplementary Materials) and Figure 1 and Figure 2 show the changes in total fatty-acid content of raw soybean flour and roasted soybean flour packed in polyethylene film and polypropylene film under different storage temperature conditions.

Except for roasted soybean flour stored at high temperature, the total fatty-acid content of flour stored at refrigeration and room temperature tended to decrease until 12 weeks and then increase for the remaining duration of storage (Figure 1). This increase is due to hydrolysis reactions that take place during storage, resulting in lipolysis and the liberation of free fatty acids [27].

The total fatty-acid content of the roasted soybean flour stored at high temperature decreased gradually between eight and 24 weeks, regardless of soybean flour type and packing material (Figure 2). At 12 weeks, the total fatty-acid content was lower than that of the initial roasted soybean flour. The decrease in fatty-acid content may be partly due to the heat treatment applied during the roasting process of the soybeans. In the autoxidation process, triplet oxygen reacts after radicals are formed by releasing hydrogen from lipid molecules. Therefore, as the temperature rises, oxidation of the roasted soybean flour, which was preheated, would be promoted, as the release of hydrogen is facilitated by the easy supply of energy to break the hydrogen bond in the lipid molecules [24]. 

Autoxidation is dependent on the ease with which the alkyl radicals of fatty acids are produced. Linolenic acid (C18:3) has more methylene groups with two double bonds, from which hydrogen atoms can be cleaved, and a higher oxidation rate than linoleic acid (C18:2) and oleic acid (C18:1) [28]. Compared to roasted soybean flour prior to storage, the major unsaturated fatty acids of the flour, i.e., oleic (C18:1), linoleic (C18:2), and linolenic acids (18:3) decreased by 30.0%, 94.4%, and 97.7%, and 38.0%, 94.8%, and 98.0% when stored in polyethylene and polypropylene film, respectively, for 48 weeks under high temperature. The secondary products of peroxide degradation of these fatty acids—hexanal, nonadienal, and 2,4-heptadienal—are used as indicator compounds for evaluating the oxidation of foods [29]. Regardless of packing material, eicosadienoic acid (C20:2) was not found at 24 weeks in roasted soybean flour stored at high temperature.

Previous studies concluded that the stability of vegetable oil depends on the type of plastic film and oxygen permeability, the level of natural antioxidants in the oil, the type and initial physicochemical properties of oil, and the storage duration and temperature. In particular, the stability was shown to differ greatly in response to different storage durations and temperatures in all types of packing materials [30]. The increase in the fatty-acid value of soybean flour of sprouted soybeans was affected by storage temperature more than humidity [31]. The relative contributions of factors studied for the retention of olive oil quality were temperature ≈ light > container headspace > packing material oxygen transmission rate [32].

Table 1 and Table 2 shows the effects of packing material, storage temperature, and storage duration on the fatty-acid composition and content of the raw and roasted soybean flour, respectively.

In this study, we showed that the most impactful of the three main factors of storage conditions was storage duration, followed by storage temperature and then packing material, regardless of the type of soybean flour. The fatty-acid composition and content of raw and roasted soybean flour, detected as C16:0, C16:1, C17:0, C18:0, C20:0, C20:3n6&21:0, and C22:0, showed considerable changes in both flour types. The fatty acids tend to change more when stored until 48 weeks than at initial storage. More changes were observed in storage duration, followed by storage temperature and packing material, as the latter two variables showed a lesser effect on fatty acids compared to storage duration. Differences were also observed in the fatty-acid composition and content sorted at different temperature based on individual components. Thus, variations between C16:0, C16:1, C17:0, C18:0, C20:0, C20:3n6&21:0, and C22:0 composition and content stored in polyethylene and polypropylene showed the effect of packing materials on fatty acids. 

The findings represent the influence of storage duration and temperature on fatty acids. Drastic variations were observed between the initial and 48-week storage of C16:0, C17:0, C18:1c&t, and C18:2c&t in raw soybean flour, whereas, in roasted soybean flour, C14:0, C16:0, C17:0, C18:1c&t, C18:2c&t, and C18:3n3 were affected. Other fatty-acid content also produced changes during storage, albeit by a lesser amount. These results showcase the impact created on each fatty-acid content of raw and roasted soybean flour which, in turn, emphasizes the importance of storage conditions. Thus, controlled storage conditions are essential for the preservation of soybean quality [33]. By controlling the soybean flour type and storage temperature conditions, and by analyzing changes in fatty-acid composition and content during long-term storage, the findings of this study further the understanding of how fatty acids can be used as indicators of quality change and storage stability in soybean flour. Furthermore, the findings of the present study provide basic data that can be used to establish the shelf life and suitable storage conditions for raw and roasted soybean flour. 

## 3. Materials and Methods

### 3.1. Soybean Material

Raw and oven-roasted (110–130 °C for 30 min) soybean flour (*Glycine max* L.) was provided by the Rural Development Administration. The soybean cultivar used was “*SaeDanbaek*”, which has a high protein content and was developed in 2010 to improve its suitability for processing tofu.

### 3.2. Storage Condition

Raw and roasted soybean flour samples were subdivided into polypropylene film (PE; 10 × 15 cm, width × length; thickness 0.05 mm) and polyethylene film (PP; 15 × 22 cm, width × length; thickness 0.04 mm) packets and immediately sealed with vinyl adhesive. The subdivided soybean flour was stored in the dark at three different temperatures: refrigeration (4 °C), room temperature (20 °C), and high temperature (45 °C, as the accelerated condition). Three packets containing each type of soybean flour, stored under each condition, were opened and used for fatty-acid analysis after 1, 2, 4, 8, 12, 24, 36, and 48 weeks of storage [34].

### 3.3. Chemicals

All solvents for fatty-acid extraction were high-performance liquid chromatography grade. Methanol (MeOH), benzene, n-heptane, 2,2-dimethoxypropane (DMP), and sulfuric acid (H_2_SO_4_) were purchased from Fisher Scientific Korea Ltd. (Seoul, Korea), Junsei Chemical Co. (Tokyo, Japan) or Daejung Chemical & Materials Co. (Gyeonggi-Do, Korea). The 37-component fatty-acid methyl ester (FAME, CRM47885) standard mixture and pentadecanoic acid (P6125) were used as internal standards, and were produced by Sigma-Aldrich Co. (St. Louis, MO, USA).

### 3.4. Sample Preparation for Fatty-Acid Measurement

Transesterification was conducted to convert the fatty acids in the soybean flour to FAMEs prior to gas chromatography/flame ionization detector (GC–FID) analysis. Soybean flour samples (50 mg) were placed in amber vials and combined with pentadecanoic acid (0.2 mg) as the internal standard. For lipid extraction and transesterification, 400 μL of n-heptane and 640 μL of methylation solvents (MeOH, benzene, DMP, and H_2_SO_4_ at 39:20:5:2, *v*/*v*/*v*/*v*) were added to the sample vials, which were capped with teflon-lined caps. The vials were shaken gently for 2 h at 60 rpm in a water bath maintained at 80 °C. The vials were then cooled to room temperature (20 °C) and centrifuged at 4000 rpm, for 1 min. The supernatant (containing FAMEs) was separated and transferred to a new container insert in the amber vial [35,36].

### 3.5. Analysis by GC–FID

The fatty-acid composition was determined using a GC–FID (Agilent 7890B, Agilent Co. Ltd., Santa Clara, CA, USA) system. A capillary column (HP-INNOWAX 19091N, 30 m × 0.25 mm, 0.25 μm, Agilent Co. Ltd., Santa Clara, CA, USA) was used to separate the 37 FAMEs. The injection volume was 1 μL with 1:50 split mode. The carrier gas was helium set at a flow rate of 10 mL∙min^−1^. The flame gas consisted of H_2_ at 35 mL∙min^−1^ and mixed gas at 300 mL∙min^−1^. The initial oven temperature was 100 °C for 2 min, later increased at the rate of 5 °C∙min^−1^ from 150 °C to 240 °C, and then held isothermally. The inlet temperature was 230 °C and the FID temperature was 250 °C. The acquisition was done from 1 to 55 min for a total analysis time of 65 min [34].

### 3.6. Quantification of Fatty Acids

The stock solution was produced by dissolving 1 mL of the 37-component FAME standard mixture in 9 mL of dichloromethane (CH_2_Cl_2_). Individual fatty acids in the sample were identified by comparing their retention times with those of the 37-component FAME standard mixture. The fatty-acid content of each sample was calculated using the analytical method specified in the Food Code issued by the Korean Food Drug Administration, as follows:

Fatty acid (mg.g^−1^) = ((P_ti_ × F_i_ × F_IS_ × Wt_IS_)/ Pt_IS_ × R_i_ × W_spl_) × 100 (1)

R_i_ = Ps_i_/Ps_IS_ × W_IS_/W_i_ (2)

where Pt_i_ is the peak area of fatty acid i, Pt_IS_ is the peak area of the internal standard, F_i_ is the conversion factor of fatty acid i, F_IS_ is the conversion factor of the internal standard, Wt_IS_ is the amount of internal standard (mg), W_spl_ is the amount of sample (g), R_i_ is the response factor of fatty acid i, Ps_i_ is the peak area of fatty acid i in the standard, Ps_IS_ is the peak area of the internal standard in the standard, W_IS_ is the amount of internal standard in the standard (mg), and W_i_ is the amount of fatty acid i in the standard (mg).

### 3.7. Statistical Analyses

Statistical analyses were performed using the general linear model of the statistical analysis program SAS (version 9.4; SAS Institute Inc., Cary, NC, USA). The experimental design and collection of samples were completely randomized, and samples were analyzed in triplicate. The significant differences of the sample means were determined using a least significant difference test at the 0.05 probability level.

## 4. Conclusions

This study determined the changes in fatty-acid content in raw and roasted soybean flour stored in different packaging materials at different temperatures for 48 weeks. Packaging material was found to have the least influence, and storage duration and temperature were found to have the greatest influence on the composition and content of fatty acids in both raw and roasted soybean flour. The present study shows the importance of selecting suitable storage temperature and duration to retain the nutritional value of soybean flour for human consumption.

## Figures and Tables

**Table 1:** Comparison of raw soybean flour fatty-acid composition and content (mg∙g^−1^) based on packaging material, storage temperature, storage duration, and the associated ANOVA *p*-values for the main factors and their interactions.

	Packing Material (P)		Storage Temperature (T)			Storage Duration (D)									*p*-Value †						
	PE	PP	REF	ROOM	HIGH	0 wk	1 wk	2 wk	4 wk	8 wk	12 wk	24 wk	36 wk	48 wk	Main factor			Interaction			
	*n* = 81	*n* = 81	*n* = 54	*n* = 54	*n* = 54	*n* = 18	*n* = 18	*n* = 18	*n* = 18	*n* = 18	*n* = 18	*n* = 18	*n* = 18	*n* = 18	P	T	D	P × T	P × D	T × D	P × T × D
C6:0	0.02	0.02	0.03	0.02	0.02	0.02	0.02	0.02	0.04	0.04	0.02	0.03	0.01	0.00	ns	ns	***	ns	ns	ns	ns
C14:0	0.14	0.14	0.15	0.14	0.14	0.17	0.19	0.18	0.13	0.13	0.10	0.12	0.13	0.14	ns	ns	****	ns	ns	ns	ns
C16:0	16.64	17.03	17.11	16.62	16.72	16.60	16.94	18.14	16.69	17.27	13.35	16.42	15.67	20.28	**	**	****	ns	***	**	ns
C16:1	0.11	0.11	0.11	0.11	0.11	0.10	0.11	0.12	0.11	0.11	0.09	0.10	0.12	0.15	ns	ns	****	ns	ns	ns	*
C17:0	0.20	0.19	0.20	0.19	0.19	0.16	0.17	0.17	0.17	0.18	0.15	0.16	0.16	0.41	*	ns	ns	*	ns	ns	**
C18:0	5.61	5.78	5.81	5.60	5.70	5.76	5.85	6.06	5.89	6.17	4.51	5.55	5.35	6.17	***	***	****	ns	****	****	ns
C18:1c&t	36.02	36.65	37.03	35.66	36.23	35.96	38.23	40.46	36.64	36.87	28.31	36.11	35.71	38.48	ns	**	****	ns	**	*	ns
C18:2c&t	69.26	71.05	71.35	69.03	68.80	64.36	73.38	77.35	70.94	73.06	59.54	67.13	68.30	73.48	**	***	****	ns	***	*	**
C18:3n3	12.06	12.21	12.38	12.15	11.87	12.07	12.74	13.04	12.17	12.51	10.65	12.02	12.00	11.98	ns	ns	****	ns	ns	ns	ns
C20:0	0.61	0.62	0.62	0.61	0.62	0.63	0.63	0.69	0.62	0.64	0.46	0.58	0.66	0.64	**	ns	****	ns	****	****	ns
C20:1n9	0.45	0.45	0.46	0.45	0.46	0.52	0.53	0.54	0.50	0.50	0.32	0.49	0.36	0.35	ns	ns	****	ns	****	ns	ns
C20:2	0.06	0.06	0.06	0.06	0.06	0.06	0.07	0.07	0.06	0.06	0.05	0.06	0.06	0.07	ns	ns	****	ns	ns	ns	ns
C20:3n6&21:0	0.08	0.08	0.08	0.08	0.08	0.08	0.09	0.09	0.08	0.08	0.06	0.08	0.08	0.09	ns	ns	****	ns	ns	ns	*
C22:0	0.99	1.00	1.01	0.98	1.00	1.02	1.03	1.10	0.98	1.00	0.73	0.96	0.98	1.17	ns	**	****	ns	****	***	**
C22:1n9	0.07	0.05	0.05	0.05	0.08	0.03	0.05	0.05	0.05	0.07	0.08	0.06	0.07	0.07	****	****	****	**	****	****	****
C23:0	0.14	0.13	0.15	0.13	0.13	0.13	0.13	0.13	0.13	0.13	0.19	0.12	0.12	0.15	****	****	****	ns	****	****	*
C24:0	0.30	0.30	0.31	0.29	0.30	0.29	0.32	0.33	0.28	0.30	0.23	0.37	0.29	0.29	ns	***	****	ns	****	*	ns
Total	142.78	145.90	146.91	142.18	142.52	137.98	150.50	158.57	145.47	149.11	118.86	140.35	140.06	153.92	**	***	****	ns	***	**	*
∑SFA	24.74	25.31	25.47	24.66	24.91	24.88	25.39	26.93	25.00	25.93	19.81	24.38	23.44	29.34	**	**	****	ns	***	***	ns
∑UFA	118.04	120.60	121.44	117.52	117.61	113.10	125.11	131.63	120.47	123.18	99.05	115.97	116.62	124.58	*	***	****	ns	***	*	**

PE: polyethylene film; PP: polypropylene film; REF: refrigeration (4 °C); ROOM: room temperature (20 °C); HIGH: high temperature (45 °C). Fatty acids are given in the format C*x*:*y*, where *x* represents the carbon chain length, and *y* represents the number of double bonds. † ns, *, **, *** and ****: non-significant, *p* < 0.05, *p* < 0.01, *p* < 0.001 and *p* < 0.0001, respectively. ∑SFA; sum of saturated fatty acids, ∑UFA; sum of unsaturated fatty acids.

**Table 2:** Comparison of roasted soybean flour fatty-acid composition and content (mg∙g^−1^) based on packaging material, storage temperature, storage duration, and the associated ANOVA *p*-value for the main factor and their interactions.

	Packing Material (P)		Storage Temperature (T)			Storage Duration (D)									*p*-Value †						
	PE	PP	REF	ROOM	HIGH	0 wk	1 wk	2 wk	4 wk	8 wk	12 wk	24 wk	36 wk	48 wk	Main factor			Interaction			
	*n* = 81	*n* = 81	*n* = 54	*n* = 54	*n* = 54	*n* = 18	*n* = 18	*n* = 18	*n* = 18	*n* = 18	*n* = 18	*n* = 18	*n* = 18	*n* = 18	P	T	D	P × T	P × D	T × D	P × T × D
C6:0	0.07	0.08	0.03	0.02	0.17	0.04	0.04	0.04	0.03	0.03	0.02	0.45	0.00	0.00	**	****	****	***	****	****	****
C14:0	0.17	0.17	0.15	0.15	0.22	0.19	0.21	0.19	0.12	0.12	0.11	0.13	0.22	0.24	*	****	****	ns	****	****	*
C16:0	16.48	16.71	16.58	16.46	16.71	16.36	17.03	17.96	15.98	17.19	13.85	16.32	15.67	18.86	ns	ns	****	ns	****	****	ns
C16:1	0.11	0.11	0.11	0.11	0.12	0.10	0.10	0.12	0.10	0.11	0.09	0.13	0.13	0.12	**	***	****	ns	**	****	ns
C17:0	0.19	0.19	0.19	0.18	0.18	0.16	0.17	0.17	0.17	0.18	0.15	0.16	0.16	0.33	ns	*	****	ns	****	****	**
C18:0	5.50	5.67	5.59	5.53	5.64	5.63	5.82	5.98	5.67	6.11	4.66	5.47	5.29	5.67	**	ns	****	ns	****	****	ns
C18:1c&t	34.24	34.86	35.83	35.45	32.55	35.33	37.82	39.77	34.87	36.61	29.47	32.86	31.96	32.77	ns	****	****	ns	****	****	ns
C18:2c&t	60.62	62.56	68.44	67.90	48.77	63.13	71.63	75.92	67.48	72.76	61.27	47.37	47.16	48.61	**	****	****	ns	****	****	ns
C18:3n3	10.53	10.88	12.06	11.99	8.32	11.89	12.35	12.79	11.59	12.46	10.93	8.43	8.28	8.43	**	****	****	ns	****	****	`ns
C20:0	0.60	0.61	0.61	0.60	0.61	0.61	0.63	0.69	0.59	0.64	0.47	0.57	0.65	0.61	**	ns	****	ns	****	****	ns
C20:1n9	0.48	0.48	0.46	0.45	0.54	0.51	0.57	0.54	0.48	0.49	0.34	0.75	0.34	0.31	ns	****	****	ns	*	****	ns
C20:2	0.05	0.06	0.06	0.06	0.04	0.05	0.07	0.06	0.06	0.06	0.05	0.04	0.04	0.04	**	****	****	ns	ns	****	ns
C20:3n6&21:0	0.09	0.09	0.08	0.08	0.09	0.08	0.09	0.09	0.07	0.08	0.07	0.08	0.10	0.11	ns	****	****	ns	**	****	ns
C22:0	0.96	0.98	0.97	0.96	1.00	0.99	1.02	1.09	0.92	0.99	0.76	0.94	0.95	1.08	**	***	****	ns	****	****	ns
C22:1n9	0.07	0.06	0.04	0.05	0.10	0.05	0.05	0.06	0.05	0.09	0.09	0.07	0.04	0.08	ns	****	**	ns	***	****	****
C23:0	0.14	0.13	0.13	0.13	0.13	0.12	0.13	0.14	0.12	0.13	0.17	0.11	0.12	0.14	***	ns	****	ns	****	**	*
C24:0	0.29	0.30	0.29	0.29	0.31	0.29	0.31	0.32	0.26	0.29	0.24	0.36	0.28	0.30	*	****	****	ns	****	****	ns
TOTAL	130.57	133.94	141.61	140.40	115.49	135.56	148.04	155.93	138.56	148.33	122.73	114.25	111.40	117.70	*	****	****	ns	****	****	ns
∑SFA	24.48	24.92	24.61	24.39	25.05	24.48	25.45	26.67	23.93	25.76	20.49	24.60	23.45	27.34	*	*	****	ns	****	****	ns
∑UFA	106.09	109.01	117.01	116.00	90.44	111.08	122.59	129.26	114.63	122.57	102.25	89.65	87.94	90.37	**	****	****	ns	****	****	ns

PE: polyethylene film, PP: polypropylene film. REF: refrigeration (4 °C), ROOM: room temperature (20 °C), HIGH: high temperature (45 °C). Fatty acids are given in the format C*x*:*y*, where *x* represents the carbon chain length, and *y* represents the number of double bonds. † ns, *, **, *** and ****: non-significant at *p* < 0.05, *p* < 0.01, *p* < 0.001 and *p* < 0.0001, respectively. ∑SFA; sum of saturated fatty acids, ∑UFA; sum of unsaturated fatty acids.

## Supplementary Materials

Steps of lipid autoxidation; Chromatograms of the 37 FAMEs; Chromatograms of the raw and roasted soybean flour prior to storage; and Chromatograms and tables of raw and roasted soybean flour packed in polyethylene and polypropylene film stored at refrigeration (4 °C), room temperature (20 °C), and high temperature (45 °C, as the accelerated condition) are available online.
