# Supplementary material for: Changes in Soybean (Glycine max L.) Flour Fatty-Acid Content Based on Storage Temperature and Duration
Source: Molecules. 2018 Oct 21;23(10):2713. doi: 10.3390/molecules23102713 (PMC6222662; doi:10.3390/molecules23102713)
Supplement: Supplementary file 1 [file molecules-23-02713-s001.pdf]

Electronic Supplementary Information (ESI) for molecules

Changes in soybean (*Glycine max* L.) flour fatty acid content based on storage temperature and duration

Mayakrishnan Prabakaran[1], Kyoung-Jin Lee[1], Yeonju An[1], Chang Kwon[1], Soyeon Kim[1], Yujin Yang[1], Ateeque Ahmad[2], Seung-Hyun Kim[1] and Ill-Min Chung[1,*]

[1]Department of Crop Science, College of Sanghuh Life Science, Konkuk University, 120 Neungdong-ro, Gwangjin-gu, Seoul 05029, Republic of Korea; prabakarannitt@gmail.com (M.P.); jin331@konkuk.ac.kr (K.J.L.); ayj3043@konkuk.ac.kr (Y.A.); chang794@konkuk.ac.kr (C.K.); hellosy1@konkuk.ac.kr (S.K.); jin9031@konkuk.ac.kr (Y.Y.); kshkim@konkuk.ac.kr (S-H.K.); imcim@konkuk.ac.kr (I.-M.C.)

[2]Process Chemistry and Technology Department, CSIR-Central Institute of Medicinal and Aromatic Plants, Lucknow 226015, India; ateeque97@gmail.com (A.A.)

*****Correspondence: imcim@konkuk.ac.kr; Tel.: +82-2-450-3730; Fax: 82-2-446-7856

Table S1. Fatty acid content of raw soybean flour packaged in polyethylene film and stored under refrigerated conditions (mg·g^{-1}, dry basis, p < 0.05, n = 3).

		Storage duration									
		Initial	1W	2W	4W	8W	12W	24W	36W	48W	LSD
	C6:0	0.02 ± 0.00^{ab}	0.03±0.00^{ab}	0.02±0.00^{ab}	0.03±0.00^{ab}	0.04±0.00^{a}	0.05±0.06^{a}	0.02±0.01^{ab}	0.02±0.00^{ab}	0.00±0.00^{b}	0.04
	C14:0	0.17±0.02^{a}	0.19±0.00^{a}	0.18±0.02^{a}	0.18±0.01^{a}	0.13±0.01^{b}	0.10±0.01^{c}	0.12±0.00^{b}	0.13±0.01^{b}	0.14±0.00^{b}	0.02
	C16:0	16.60±0.18^{cd}	16.38±0.17^{de}	17.06±0.56^{cd}	18.80±0.72^{b}	17.47±0.03^{c}	14.03±1.34^{f}	16.22±0.21^{de}	15.59±0.15^{e}	20.58±0.30^{a}	0.96
	C16:1	0.10±0.00^{c}	0.11±0.02^{bc}	0.09±0.01^{c}	0.13±0.00^{ab}	0.11±0.00^{bc}	0.11±0.03^{bc}	0.10±0.00^{c}	0.12±0.01^{bc}	0.15±0.02^{a}	0.03
	C17:0	0.16±0.01^{b}	0.17±0.00^{b}	0.17±0.01^{b}	0.19±0.01^{b}	0.18±0.01^{b}	0.15±0.01^{b}	0.17±0.00^{b}	0.16±0.00^{b}	0.52±0.09^{a}	0.05
	C18:0	5.76±0.07^{cd}	5.63±0.06^{d}	5.69±0.20^{d}	6.59±0.25^{a}	6.06±0.12^{bc}	4.67±0.41^{f}	5.48±0.05^{de}	5.25±0.04^{e}	6.31±0.11^{ab}	0.32
	C18:1c&t	35.96±0.41^{d}	36.66±0.48^{cd}	37.99±1.36^{d}	41.56±1.62^{a}	38.15±0.06^{bc}	29.89±2.67^{e}	35.79±0.36^{d}	35.08±0.32^{d}	38.91±0.43^{b}	2.02
	C18:2c&t	64.36±0.94^{ef}	69.54±2.24^{cd}	72.61±2.64^{bc}	80.88±3.10^{a}	73.88±0.18^{bc}	63.11±5.83^{f}	67.20±1.45^{def}	67.61±0.66^{de}	75.51±0.79^{b}	4.41
	C18:3n3	12.07±0.19^{bc}	11.97±0.59^{bc}	12.26±0.48^{b}	13.98±0.55^{a}	12.66±0.05^{b}	11.34±1.03^{c}	12.22±0.43^{b}	12.12±0.13^{bc}	13.56±0.15^{a}	0.85
Refrigeration	C20:0	0.63±0.01^{b}	0.61±0.01^{b}	0.65±0.02^{b}	0.70±0.03^{a}	0.65±0.01^{b}	0.49±0.04^{d}	0.57±0.01^{c}	0.62±0.04^{b}	0.64±0.01^{b}	0.04
(4℃)	C20:1n9	0.52±0.04^{ab}	0.55±0.04^{a}	0.51±0.02^{ab}	0.53±0.04^{ab}	0.54±0.01^{a}	0.33±0.03^{c}	0.47±0.05^{b}	0.35±0.03^{c}	0.35±0.00^{c}	0.05
	C20:2	0.06±0.01^{c}	0.06±0.00^{abc}	0.07±0.00^{ab}	0.07±0.00^{ab}	0.07±0.00^{a}	0.06±0.01^{bc}	0.06±0.01^{c}	0.06±0.00^{bc}	0.07±0.00^{ab}	0.01
	C20:3n6&21:0	0.08±0.01^{cd}	0.09±0.01^{abc}	0.09±0.01^{ab}	0.09±0.01^{a}	0.08±0.01^{abcd}	0.07±0.02^{d}	0.08±0.00^{cd}	0.08±0.00^{bcd}	0.09±0.01^{ab}	0.01
	C22:0	1.02±0.02^{c}	0.99±0.01^{cde}	1.04±0.05^{c}	1.11±0.06^{b}	1.02±0.01^{cd}	0.78±0.07^{f}	0.95±0.01^{e}	0.95±0.02^{de}	1.24±0.02^{a}	0.06
	C22:1n9	0.03±0.01^{e}	0.06±0.01^{bc}	0.05±0.02^{cd}	0.06±0.00^{bc}	0.06±0.01^{bc}	0.08±0.01^{a}	0.05±0.01^{bcd}	0.04±0.01^{de}	0.06±0.00^{b}	0.01
	C23:0	0.13±0.01^{cd}	0.13±0.01^{d}	0.13±0.01^{cd}	0.16±0.02^{b}	0.13±0.00^{cd}	0.30±0.04^{a}	0.12±0.01^{d}	0.12±0.00^{d}	0.15±0.01^{cb}	0.03
	C24:0	0.29±0.01^{bc}	0.31±0.01^{bc}	0.31±0.02^{bc}	0.32±0.02^{b}	0.30±0.01^{bc}	0.24±0.03^{d}	0.36±0.02^{a}	0.29±0.00^{c}	0.32±0.01^{b}	0.03
	Total	137.98±1.75^{d}	143.48±3.25^{cd}	148.93±5.41^{c}	165.37±6.40^{a}	151.51±0.25^{bc}	125.80±11.47^{e}	139.98±2.19^{d}	138.58±1.38^{d}	158.59±1.59^{ab}	8.57
	∑SFA	24.88±0.24^{cd}	24.53±0.23^{de}	25.36±0.90^{cd}	28.17±1.11^{b}	26.05±0.16^{c}	20.88±1.91^{f}	24.09±0.26^{de}	23.20±0.26^{e}	29.99±0.36^{a}	1.41
	∑UFA	113.10±1.51^{d}	118.95±3.21^{cd}	123.57±4.51^{bc}	137.20±5.30^{a}	125.46±0.21^{bc}	104.92±9.56^{e}	115.89±2.10^{d}	115.38±1.12^{d}	128.60±1.36^{b}	7.23

∑SFA; sum of saturated fatty acids, ∑UFA; sum of unsaturated fatty acids.

Table S2. Fatty acid content of raw soybean flour packaged in polyethylene film and stored under room temperature conditions (mg·g^{-1}, dry basis, $p < 0.05$, n = 3).

		Storage duration									
		Initial	1W	2W	4W	8W	12W	24W	36W	48W	LSD
Room temperature (20℃)	C6:0	0.02±0.00^{b}	0.03±0.00^{b}	0.02±0.00^{b}	0.03±0.00^{b}	0.04±0.00^{a}	0.02±0.00^{c}	0.03±0.00^{b}	0.02±0.01^{b}	0.00±0.00^{d}	0.01
	C14:0	0.17±0.02^{a}	0.19±0.03^{a}	0.18±0.01^{a}	0.13±0.01^{b}	0.13±0.00^{b}	0.10±0.00^{c}	0.12±0.01^{bc}	0.12±0.00^{bc}	0.14±0.00^{b}	0.02
	C16:0	16.60±0.18^{bcd}	16.52±0.24^{cd}	17.85±2.07^{b}	16.23±0.86^{cd}	17.24±0.15^{bc}	12.79±0.17^{e}	16.04±0.02^{cd}	15.41±0.14^{d}	20.22±0.38^{a}	1.32
	C16:1	0.10±0.00^{bc}	0.11±0.01^{bc}	0.14±0.03^{a}	0.11±0.01^{bc}	0.10±0.00^{bc}	0.09±0.02^{c}	0.10±0.00^{bc}	0.12±0.01^{ab}	0.14±0.01^{a}	0.03
	C17:0	0.16±0.01^{c}	0.17±0.01^{bc}	0.17±0.01^{bc}	0.16±0.01^{c}	0.18±0.00^{b}	0.15±0.01^{d}	0.16±0.00^{cd}	0.16±0.00^{cd}	0.40±0.01^{a}	0.01
	C18:0	5.76±0.07^{abcd}	5.71±0.09^{bcd}	5.97±0.69^{abc}	5.57±0.29^{cde}	6.07±0.08^{ab}	4.25±0.08^{f}	5.41±0.02^{de}	5.16±0.06^{e}	6.17±0.14a	0.45
	C18:1c&t	35.96±0.41^{bc}	36.88±1.07^{abc}	39.93±4.86^{a}	35.91±1.88^{bc}	37.54±0.43^{abc}	27.03±0.46^{d}	35.34±0.13^{bc}	34.60±0.30^{c}	38.19±0.32^{ab}	3.08
	C18:2c&t	64.36±0.94^{d}	69.49±4.34^{bcd}	76.20±9.64^{a}	69.84±3.71^{abcd}	72.59±0.91^{abc}	57.07±1.18^{e}	66.42±0.47^{cd}	66.55±0.48^{cd}	73.33±0.80^{ab}	6.51
	C18:3n3	12.07±0.19^{a}	11.90±0.96^{a}	12.86±1.74^{a}	12.04±0.64^{a}	12.45±0.18^{a}	10.24±0.23^{b}	12.00±0.14^{a}	11.84±0.05^{a}	12.92±0.22^{a}	1.22
	C20:0	0.63±0.01^{ab}	0.61±0.02^{bc}	0.67±0.08^{a}	0.61±0.04^{bc}	0.64±0.01^{ab}	0.43±0.01^{d}	0.56±0.01^{c}	0.64±0.01^{ab}	0.63±0.03^{ab}	0.06
	C20:1n9	0.52±0.04^{ab}	0.55±0.04^{a}	0.52±0.05^{ab}	0.46±0.02^{c}	0.53±0.01^{a}	0.31±0.01^{e}	0.48±0.01^{bc}	0.36±0.00^{d}	0.32±0.02^{de}	0.05
	C20:2	0.06±0.01^{ab}	0.06±0.02^{ab}	0.07±0.01^{a}	0.06±0.01^{ab}	0.06±0.00^{ab}	0.05±0.00^{b}	0.06±0.00^{ab}	0.06±0.00^{ab}	0.07±0.00^{a}	0.01
	C20:3n6&21:0	0.08±0.01^{bc}	0.09±0.02^{a}	0.09±0.01^{a}	0.07±0.01^{c}	0.07±0.01^{bc}	0.06±0.00^{c}	0.07±0.01^{c}	0.08±0.00^{bc}	0.09±0.00^{ab}	0.02
	C22:0	1.02±0.02^{bc}	0.99±0.02^{cd}	1.08±0.11^{b}	0.95±0.05^{d}	0.99±0.00^{cd}	0.72±0.01^{e}	0.93±0.00^{d}	0.96±0.03^{cd}	1.17±0.02^{a}	0.07
	C22:1n9	0.03±0.01^{c}	0.06±0.01^{b}	0.05±0.01^{b}	0.06±0.00^{b}	0.06±0.00^{b}	0.09±0.00^{a}	0.07±0.02^{b}	0.06±0.02^{b}	0.09±0.01^{a}	0.02
	C23:0	0.13±0.01^{c}	0.13±0.01^{cd}	0.13±0.01^{cd}	0.11±0.01^{f}	0.13±0.01^{cde}	0.21±0.01^{a}	0.12±0.00^{def}	0.12±0.00^{ef}	0.15±0.01^{b}	0.01
	C24:0	0.29±0.01^{cd}	0.32±0.01^{bc}	0.33±0.04^{ab}	0.28±0.01^{d}	0.28±0.02^{d}	0.23±0.01^{e}	0.35±0.00^{a}	0.29±0.00^{d}	0.30±0.01^{cd}	0.03
	Total	137.98±1.75^{c}	143.82±6.67^{abc}	156.27±19.30^{a}	142.61±7.52^{bc}	149.10±1.71^{abc}	113.85±2.14^{d}	138.25±0.79^{c}	136.53±1.05^{c}	154.34±1.73^{ab}	12.64
	∑SFA	24.88±0.24^{bcd}	24.76±0.33^{bcde}	26.51±3.03^{b}	24.13±1.26^{bde}	25.77±0.22^{bv}	18.96±0.26^{f}	23.79±0.06^{de}	22.94±0.22^{e}	29.28±0.57^{a}	1.93
	∑UFA	113.10±1.51^{c}	119.05±6.35^{abc}	129.77±16.28^{a}	118.48±6.26^{bc}	123.34±1.52^{abc}	94.88±1.89^{d}	114.47±0.74^{bc}	113.59±0.83^{c}	125.06±1.30^{ab}	10.79

∑SFA; sum of saturated fatty acids, ∑UFA; sum of unsaturated fatty acids.

Table S3. Fatty acid content of raw soybean flour packaged in polyethylene film and stored under high temperature conditions (mg·g^{-1}, dry basis, p < 0.05, n = 3).

		Storage duration									
		Initial	1W	2W	4W	8W	12W	24W	36W	48W	LSD
	C6:0	0.02±0.00^{c}	0.02±0.00^{c}	0.02±0.00^{c}	0.02±0.00^{cd}	0.04±0.01^{a}	0.02±0.00^{d}	0.03±0.01^{d}	0.02±0.00^{cd}	0.00±0.00^{e}	0.01
	C14:0	0.17±0.02^{a}	0.18±0.02^{a}	0.18±0.03^{a}	0.11±0.01^{cd}	0.13±0.01^{bc}	0.11±0.00^{d}	0.12±0.01^{bcd}	0.13±0.01^{bcd}	0.14±0.00^{b}	0.02
	C16:0	16.60±0.18^{b}	16.81±0.04^{b}	16.95±0.47^{b}	14.23±0.17^{c}	17.71±0.21^{b}	13.92±0.31^{c}	16.56±0.22^{b}	14.13±3.08^{c}	20.73±0.27^{a}	1.81
	C16:1	0.10±0.00^{de}	0.12±0.02^{b}	0.11±0.01^{cd}	0.09±0.00^{e}	0.10±0.01^{de}	0.09±0.00^{e}	0.10±0.00^{de}	0.12±0.01^{bc}	0.15±0.00^{a}	0.01
	C17:0	0.16±0.01^{cd}	0.17±0.00^{bc}	0.16±0.01^{cd}	0.15±0.00^{e}	0.18±0.00^{b}	0.16±0.01^{cd}	0.15±0.02^{ed}	0.16±0.00^{ed}	0.41±0.01^{a}	0.01
	C18:0	5.76±0.07^{bc}	5.81±0.02^{b}	5.65±0.15^{bc}	4.88±0.06^{e}	6.25±0.19^{a}	4.66±0.10^{f}	5.62±0.06^{c}	5.31±0.05^{d}	6.38±0.12^{a}	0.18
	C18:1c&t	35.96±0.41^{bc}	38.01±0.10^{a}	37.75±1.14^{a}	31.24±0.46^{d}	38.37±0.68^{a}	29.71±0.86^{e}	36.66±0.30^{b}	35.36±0.38^{c}	38.09±0.33^{a}	1.03
	C18:2c&t	64.36±0.94^{cd}	73.28±0.16^{a}	71.88±2.73^{a}	60.11±1.31^{e}	72.35±2.08^{a}	62.08±1.57^{de}	67.75±1.20^{b}	66.79±1.00^{bc}	70.98±0.92^{a}	2.57
	C18:3n3	12.07±0.19^{b}	12.72±0.01^{a}	12.07±0.54^{b}	10.24±0.25^{e}	12.09±0.45^{b}	11.00±0.29^{d}	11.90±0.33^{bc}	11.37±0.18^{cd}	11.63±0.28^{bc}	0.54
High temperature (45℃)	C20:0	0.63±0.01^{b}	0.63±0.01^{b}	0.64±0.02^{ab}	0.53±0.01^{d}	0.65±0.01^{ab}	0.48±0.01^{e}	0.58±0.01^{c}	0.65±0.04^{ab}	0.67±0.01^{a}	0.03
	C20:1n9	0.52±0.04^{ab}	0.52±0.03^{ab}	0.53±0.03^{ab}	0.45±0.01^{c}	0.57±0.05^{a}	0.33±0.02^{d}	0.49±0.02^{bc}	0.35±0.04^{d}	0.34±0.05^{d}	0.06
	C20:2	0.06±0.01^{bc}	0.07±0.01^{a}	0.06±0.01^{ab}	0.05±0.00^{c}	0.06±0.01^{ab}	0.06±0.01^{bc}	0.06±0.00^{abc}	0.06±0.00^{abc}	0.07±0.00^{ab}	0.01
	C20:3n6&21:0	0.08±0.01^{cd}	0.09±0.01^{a}	0.09±0.01^{ab}	0.07±0.01^{de}	0.08±0.00^{abc}	0.06±0.00^{e}	0.08±0.00^{c}	0.08±0.00^{bc}	0.09±0.00^{a}	0.01
	C22:0	1.02±0.02^{b}	1.03±0.01^{b}	1.02±0.04^{b}	0.83±0.01^{d}	1.02±0.02^{b}	0.77±0.02^{e}	0.97±0.01^{c}	0.99±0.03^{bc}	1.26±0.03^{a}	0.04
	C22:1n9	0.03±0.01^{e}	0.06±0.01^{d}	0.06±0.01^{d}	0.05±0.00^{de}	0.07±0.01^{d}	0.12±0.04^{c}	0.07±0.00^{d}	0.15±0.02^{b}	0.21±0.02^{a}	0.03
	C23:0	0.13±0.01^{b}	0.13±0.00^{bc}	0.12±0.01^{bc}	0.10±0.00^{c}	0.12±0.01^{bc}	0.21±0.06^{a}	0.12±0.00^{bc}	0.12±0.01^{bc}	0.15±0.01^{b}	0.03
	C24:0	0.29±0.01^{c}	0.32±0.00^{b}	0.31±0.01^{bc}	0.24±0.01^{d}	0.31±0.01^{bc}	0.24±0.00^{d}	0.37±0.01^{a}	0.30±0.01^{c}	0.32±0.01^{b}	0.02
	Total	137.98±1.75^{bc}	149.99±0.39^{a}	147.61±5.08^{a}	123.39±2.26^{d}	150.11±3.32^{a}	124.01±3.18^{d}	141.64±2.02^{b}	136.08±4.63^{c}	151.62±1.90^{a}	5.25
	∑SFA	24.88±0.24^{b}	25.20±0.08^{b}	25.14±0.71^{b}	21.15±0.24^{c}	26.50±0.37^{b}	20.62±0.42^{c}	24.61±0.31^{b}	21.88±3.12^{c}	30.15±0.44^{a}	1.90
	∑UFA	113.10±1.51^{b}	124.79±0.31^{a}	122.47±4.40^{a}	102.24±2.02^{c}	123.62±3.14^{a}	103.39±2.76^{c}	117.03±1.76^{b}	114.20±1.53^{b}	121.46±1.47^{a}	4.08

∑SFA; sum of saturated fatty acids, ∑UFA; sum of unsaturated fatty acids.

Table S4. Fatty acid content of roasted soybean flour packaged in polyethylene film and stored under refrigerated conditions (mg·g^{-1}, dry basis, p < 0.05, n = 3).

		Storage duration									
		Initial	1W	2W	4W	8W	12W	24W	36W	48W	LSD
	C6:0	0.04±0.00^{bc}	0.05±0.02^{a}	0.04±0.01^{bc}	0.04±0.01^{b}	0.03±0.00^{c}	0.02±0.00^{d}	0.03±0.00^{c}	0.00±0.00^{e}	0.00±0.00^{e}	0.01
	C14:0	0.19±0.02^{b}	0.24±0.02^{a}	0.18±0.03^{b}	0.11±0.02^{c}	0.12±0.01^{c}	0.11±0.02^{c}	0.13±0.00^{c}	0.12±0.00^{c}	0.13±0.00^{c}	0.03
	C16:0	16.36±0.21^{b}	16.85±0.15^{b}	15.48±3.47^{bc}	15.33±2.11^{bc}	17.11±0.19^{b}	13.89±0.45^{c}	16.36±0.05^{b}	16.03±0.18^{bc}	20.74±0.20^{a}	2.35
	C16:1	0.10±0.00^{bc}	0.10±0.00^{bc}	0.09±0.02^{c}	0.10±0.01^{bc}	0.11±0.00^{bc}	0.09±0.00^{c}	0.10±0.00^{bc}	0.12±0.01^{ab}	0.13±0.02^{a}	0.02
	C17:0	0.16±0.01^{bcd}	0.17±0.00^{bc}	0.15±0.03^{d}	0.16±0.02^{bcd}	0.18±0.00^{b}	0.15±0.01^{cd}	0.17±0.00^{bcd}	0.16±0.00^{bcd}	0.38±0.01^{a}	0.02
	C18:0	5.63±0.07^{abc}	5.73±0.05^{abc}	5.16±1.16^{cd}	5.33±0.77^{bcd}	6.00±0.09^{ab}	4.65±0.13^{d}	5.48±0.03^{abc}	5.32±0.05^{bcd}	6.25±0.08^{a}	0.81
	C18:1c&t	35.33±0.47^{ab}	36.48±0.58^{ab}	34.23±7.89^{bc}	33.58±4.55^{bc}	37.69±0.46^{ab}	29.51±1.09^{c}	35.74±0.31^{ab}	35.55±0.48^{ab}	39.54±0.13a	5.28
	C18:2c&t	63.13±0.83^{b}	65.22±3.68^{ab}	65.03±16.02^{ab}	65.29±9.21^{ab}	72.89±0.88^{ab}	62.33±2.15^{b}	67.36±1.27^{ab}	67.90±1.21^{ab}	75.52±0.30^{a}	10.91
	C18:3n3	11.89±0.16^{ab}	10.91±0.81^{b}	10.90±2.80^{b}	11.27±1.65^{b}	12.49±0.16^{ab}	11.28±0.38^{b}	12.26±0.36^{ab}	12.17±0.26^{ab}	13.32±0.10^{a}	1.95
Refrigeration	C20:0	0.61±0.00^{ab}	0.61±0.00^{ab}	0.60±0.14^{ab}	0.57±0.07^{b}	0.64±0.01^{ab}	0.47±0.02^{c}	0.56±0.00^{bc}	0.69±0.01^{a}	0.64±0.01^{ab}	0.09
(4℃)	C20:1n9	0.51±0.03^{b}	0.63±0.04^{bc}	0.48±0.07^{bc}	0.44±0.04^{c}	0.51±0.03^{b}	0.34±0.01^{d}	0.49±0.03^{bc}	0.37±0.01^{d}	0.35±0.01^{d}	0.06
	C20:2	0.05±0.01^{b}	0.05±0.01^{b}	0.05±0.01^{ab}	0.06±0.00^{ab}	0.06±0.01^{a}	0.05±0.00^{ab}	0.06±0.01^{ab}	0.06±0.00^{ab}	0.07±0.00^{a}	0.01
	C20:3n6&21:0	0.08±0.01^{bc}	0.10±0.03^{bc}	0.08±0.01^{bc}	0.07±0.01^{bc}	0.08±0.01^{bc}	0.07±0.00^{c}	0.08±0.00^{bc}	0.08±0.00^{bc}	0.09±0.00^{ab}	0.02
	C22:0	0.99±0.01^{ab}	0.99±0.01^{b}	0.94±0.22^{b}	0.87±0.11^{bc}	1.01±0.02^{ab}	0.75±0.04^{c}	0.94±0.01^{b}	0.91±0.01^{b}	1.13±0.02^{a}	0.14
	C22:1n9	0.05±0.01^{b}	0.05±0.02^{b}	0.06±0.01^{b}	0.05±0.01^{b}	0.05±0.01^{b}	0.08±0.00^{a}	0.05±0.01^{b}	0.00±0.00^{c}	0.05±0.00^{b}	0.02
	C23:0	0.12±0.00^{b}	0.12±0.00^{b}	0.12±0.03^{b}	0.11±0.03^{b}	0.13±0.01^{b}	0.26±0.05^{a}	0.12±0.00^{b}	0.12±0.00^{b}	0.14±0.00^{b}	0.04
	C24:0	0.29±0.01^{b}	0.29±0.00^{b}	0.28±0.06^{bc}	0.25±0.03^{c}	0.29±0.01^{bc}	0.25±0.01^{c}	0.36±0.00^{a}	0.27±0.00^{bc}	0.29±0.00^{b}	0.04
	Total	135.56±1.77^{bc}	138.60±4.93^{abc}	133.87±31.95^{bc}	133.64±18.64^{bc}	149.39±1.60^{ab}	124.31±4.24^{c}	140.29±1.99^{abc}	139.87±2.20^{abc}	158.76±0.72^{a}	21.59
	∑SFA	24.48±0.30^{b}	25.16±0.23^{b}	23.01±5.14^{bc}	22.85±3.17^{bc}	25.58±0.15^{b}	20.62±0.62^{c}	24.22±0.09^{b}	23.70±0.24^{bc}	29.79±0.31^{a}	3.49
	∑UFA	111.08±1.47^{abc}	113.44±5.01^{abc}	110.85±26.81^{abc}	110.80±15.47^{bc}	123.81±1.50^{ab}	103.69±3.62^{c}	116.06±1.91^{abc}	116.17±1.96^{abc}	128.97±0.45^{a}	18.16

∑SFA; sum of saturated fatty acids, ∑UFA; sum of unsaturated fatty acids.

Table S5. Fatty acid content of roasted soybean flour packaged in polyethylene film and stored under room temperature conditions ($mg \cdot g^{-1}$, dry basis, $p < 0.05$, n = 3).

		Storage duration									
		Initial	1W	2W	4W	8W	12W	24W	36W	48W	LSD
Room temperature (20℃)	C6:0	0.04±0.00^{a}	0.03±0.02^{ab}	0.04±0.00^{a}	0.04±0.01^{a}	0.03±0.00^{ab}	0.02±0.00^{b}	0.03±0.00^{ab}	0.00±0.00^{c}	0.00±0.00^{c}	0.02
	C14:0	0.19±0.02^{b}	0.23±0.02^{a}	0.19±0.00^{b}	0.11±0.02^{cd}	0.12±0.00^{c}	0.10±0.00^{d}	0.12±0.00^{c}	0.12±0.01^{cd}	0.13±0.00^{c}	0.02
	C16:0	16.36±0.21^{bc}	16.81±0.09^{b}	16.71±0.08^{b}	14.47±3.54^{cd}	16.99±0.07^{b}	13.14±0.26^{d}	16.28±0.02^{bc}	15.90±0.10^{bc}	20.39±0.14^{a}	2.04
	C16:1	0.10±0.00^{bcd}	0.10±0.00^{bcd}	0.10±0.01^{bcd}	0.09±0.02^{cd}	0.12±0.02^{ab}	0.08±0.00^{d}	0.10±0.00^{bcd}	0.12±0.02^{abc}	0.14±0.02^{a}	0.02
	C17:0	0.16±0.01^{bc}	0.17±0.00^{b}	0.16±0.01^{bc}	0.15±0.03^{c}	0.18±0.00^{b}	0.14±0.01^{c}	0.17±0.00^{bc}	0.16±0.00^{bc}	0.36±0.01^{a}	0.02
	C18:0	5.63±0.07^{abc}	5.72±0.04^{ab}	5.55±0.01^{abc}	4.96±1.21^{cd}	5.93±0.15^{ab}	4.38±0.12^{d}	5.45±0.01^{bc}	5.29±0.05^{bc}	6.18±0.04^{a}	0.70
	C18:1c&t	35.33±0.47^{ab}	37.12±0.11^{a}	36.94±0.15^{a}	31.91±8.14^{bc}	37.32±0.16^{a}	27.73±0.72^{c}	35.63±0.03^{ab}	35.28±0.31^{ab}	38.65±0.57^{a}	4.70
	C18:2c&t	63.13±0.83^{bcd}	69.76±0.81^{abc}	70.66±0.12^{abc}	62.03±16.00^{cd}	72.21±0.36^{ab}	58.48±1.56^{d}	67.38±0.23^{abcd}	67.27±0.72^{abcd}	73.47±1.47^{a}	9.27
	C18:3n3	11.89±0.16^{abc}	11.94±0.23^{abc}	11.92±0.06^{abc}	10.73±2.85^{bc}	12.35±0.06^{ab}	10.58±0.31^{c}	12.34±0.11^{ab}	12.03±0.14^{abc}	12.95±0.35^{a}	1.66
	C20:0	0.61±0.00^{ab}	0.62±0.01^{ab}	0.63±0.00^{ab}	0.53±0.13^{c}	0.62±0.01^{ab}	0.44±0.02^{d}	0.56±0.00^{bc}	0.68±0.01^{a}	0.61±0.01^{b}	0.08
	C20:1n9	0.51±0.03^{b}	0.59±0.01^{a}	0.50±0.02^{b}	0.41±0.08^{cd}	0.51±0.01^{b}	0.31±0.03^{e}	0.47±0.01^{bc}	0.37±0.00^{de}	0.32±0.02^{e}	0.06
	C20:2	0.05±0.01^{c}	0.07±0.01^{a}	0.06±0.01^{ab}	0.05±0.02^{bc}	0.06±0.00^{abc}	0.05±0.00^{bc}	0.06±0.00^{abc}	0.06±0.00^{abc}	0.06±0.00^{ab}	0.01
	C20:3n6&21:0	0.08±0.01^{cd}	0.10±0.00^{a}	0.08±0.02^{bc}	0.07±0.01^{cd}	0.08±0.00^{abc}	0.06±0.01^{d}	0.07±0.00^{cd}	0.08±0.00^{bc}	0.09±0.01^{ab}	0.02
	C22:0	0.99±0.01^{b}	0.99±0.01^{b}	1.02±0.01^{ab}	0.84±0.20^{c}	0.98±0.01^{b}	0.71±0.00^{d}	0.94±0.00^{bc}	0.91±0.02^{bc}	1.14±0.01^{a}	0.12
	C22:1n9	0.05±0.01^{bc}	0.06±0.01^{bc}	0.06±0.01^{b}	0.05±0.01^{bc}	0.05±0.01^{bc}	0.09±0.02^{a}	0.07±0.00^{b}	0.00±0.00^{d}	0.04±0.01^{c}	0.01
	C23:0	0.12±0.00^{bc}	0.13±0.01^{bc}	0.13±0.01^{bc}	0.10±0.02^{c}	0.13±0.00^{bc}	0.26±0.04^{a}	0.12±0.00^{c}	0.11±0.00^{c}	0.15±0.02^{b}	0.03
	C24:0	0.29±0.01^{bc}	0.29±0.01^{bc}	0.30±0.01^{b}	0.24±0.06^{de}	0.30±0.01^{b}	0.22±0.01^{e}	0.36±0.01^{a}	0.26±0.01^{cd}	0.29±0.00^{bc}	0.04
	Total	135.56±1.77^{bc}	144.72±0.79^{abc}	145.06±0.24^{abc}	126.79±32.37^{cd}	147.99±0.70^{ab}	116.80±3.04^{d}	140.15±0.31^{abc}	138.63±1.31^{abc}	154.98±2.50^{a}	18.70
	∑SFA	24.48±0.30^{bc}	25.08±0.17^{b}	24.81±0.10^{b}	21.50±5.25^{cd}	25.37±0.23^{b}	19.47±0.44^{d}	24.10±0.01^{bc}	23.51±0.16^{bc}	29.34±0.22^{a}	3.02
	∑UFA	111.08±1.47^{abc}	119.64±0.97^{ab}	120.24±0.17^{ab}	105.29±27.12^{bc}	122.63±0.59^{a}	97.32±2.60^{c}	116.05±0.32^{ab}	115.12±1.15^{ab}	125.64±2.40^{a}	15.69

∑SFA; sum of saturated fatty acids, ∑UFA; sum of unsaturated fatty acids.

Table S6. Fatty acid content of roasted soybean flour packaged in polyethylene film and stored under high temperature conditions (mg·g^{-1}, dry basis, p < 0.05, n = 3).

		Storage duration								
		Initial	1W	2W	4W	8W	12W	24W	36W	48W	LSD
	C6:0	0.04±0.00^{b}	0.04±0.01^{b}	0.04±0.00^{b}	0.04±0.00^{b}	0.03±0.00^{b}	0.02±0.00^{b}	1.09±0.24^{a}	0.00±0.00^{b}	0.00±0.00^{b}	0.14
	C14:0	0.19±0.02^{c}	0.21±0.03^{c}	0.19±0.02^{c}	0.12±0.01^{de}	0.13±0.02^{d}	0.10±0.00^{e}	0.12±0.01^{de}	0.42±0.00^{b}	0.48±0.01^{a}	0,03
	C16:0	16.36±0.21^{cd}	17.66±1.26^{ab}	17.48±0.28^{b}	17.06±0.68^{bc}	17.42±0.03^{b}	14.30±0.04^{e}	16.01±0.05^{d}	14.83±0.38^{e}	18.49±0.13^{a}	0.87
	C16:1	0.10±0.00^{c}	0.11±0.01^{bc}	0.11±0.01^{bc}	0.11±0.00^{bc}	0.11±0.01^{bc}	0.09±0.00^{c}	0.17±0.03^{a}	0.12±0.01^{b}	0.10±0.00^{c}	0.02
	C17:0	0.16±0.01^{cdef}	0.18±0.02^{bc}	0.17±0.00^{bcde}	0.17±0.01^{bcd}	0.18±0.00^{b}	0.16±0.00^{ef}	0.16±0.00^{def}	0.15±0.00^{f}	0.34±0.01^{a}	0.01
	C18:0	5.63±0.07^{bcd}	6.02±0.42^{a}	5.82±0.08^{abc}	5.96±0.45^{ab}	6.12±0.20^{a}	4.85±0.00^{e}	5.36±0.02^{d}	4.87±0.14^{e}	5.51±0.04^{cd}	0.38
	C18:1c&t	35.33±0.47^{b}	39.32±2.95^{a}	38.60±0.60^{a}	37.36±1.58^{ab}	38.25±0.18^{a}	30.40±0.30^{c}	26.24±0.35^{d}	22.88±0.82^{e}	24.72±0.33^{de}	2.05
	C18:2c&t	63.13±0.83^{b}	75.24±6.52^{a}	73.33±1.33^{a}	71.83±3.17^{a}	73.92±0.45^{a}	61.92±0.10^{b}	6.07±0.60^{c}	3.60±0.15^{c}	3.56±0.11^{c}	4.27
	C18:3n3	11.89±0.16^{b}	13.05±1.24^{a}	12.30±0.26^{ab}	12.25±0.58^{ab}	12.56±0.10^{ab}	10.74±0.09^{c}	0.49±0.03^{d}	0.25±0.02^{d}	0.27±0.01^{d}	0.81
High temperature (45℃)	C20:0	0.61±0.00^{c}	0.65±0.05^{abc}	0.67±0.02^{a}	0.63±0.04^{bc}	0.64±0.00^{abc}	0.48±0.00^{e}	0.56±0.01^{d}	0.57±0.01^{d}	0.66±0.00^{ab}	0.04
	C20:1n9	0.51±0.03^{bc}	0.57±0.08^{b}	0.55±0.02^{b}	0.53±0.01^{b}	0.50±0.00^{bc}	0.34±0.01^{cd}	1.25±0.29^{a}	0.25±0.02^{d}	0.31±0.00^{d}	0.17
	C20:2	0.05±0.01^{b}	0.07±0.01^{a}	0.06±0.01^{ab}	0.06±0.00^{ab}	0.06±0.01^{ab}	0.06±0.00^{b}	0.00±0.00^{c}	0.00±0.00^{c}	0.00±0.00^{c}	0.01
	C20:3n6&21:0	0.08±0.01^{cde}	0.10±0.01^{b}	0.09±0.00^{bc}	0.09±0.01^{bc}	0.08±0.00^{cd}	0.07±0.00^{e}	0.07±0.00^{de}	0.14±0.00^{a}	0.14±0.00^{a}	0.01
	C22:0	0.99±0.01^{c}	1.05±0.07^{bc}	1.06±0.01^{b}	1.00±0.04^{c}	1.01±0.01^{bc}	0.79±0.00^{e}	0.91±0.02^{d}	0.93±0.06^{d}	1.17±0.03^{a}	0.06
	C22:1n9	0.05±0.01^{bc}	0.06±0.01^{abc}	0.07±0.01^{abc}	0.06±0.01^{abc}	0.22±0.27^{a}	0.14±0.03^{abc}	0.11±0.04^{abc}	0.00±0.00^{c}	0.20±0.09^{ab}	0.17
	C23:0	0.12±0.00^{bc}	0.13±0.01^{bc}	0.13±0.00^{bc}	0.12±0.01^{bc}	0.12±0.01^{bc}	0.17±0.06^{a}	0.10±0.00^{c}	0.13±0.01^{bc}	0.14±0.01^{b}	0.03
	C24:0	0.29±0.01^{bc}	0.31±0.02^{bc}	0.32±0.01^{b}	0.29±0.02^{c}	0.30±0.00^{bc}	0.25±0.01^{d}	0.36±0.01^{a}	0.29±0.03^{bc}	0.39±0.02^{a}	0.03
	Total	135.56±1.77^{b}	154.76±12.49^{a}	150.96±2.59^{a}	147.67±6.56^{a}	151.66±0.40^{a}	124.91±0.41^{c}	59.08±0.78^{d}	49.43±1.63^{e}	56.48±0.73^{de}	8.34
	∑SFA	24.48±0.30^{d}	26.34±1.88^{ab}	25.96±0.41^{abc}	25.48±1.23^{bcd}	26.03±0.24^{abc}	21.20±0.03^{e}	24.75±0.19^{cd}	22.32±0.61^{e}	27.32±0.19^{a}	1.37
	∑UFA	111.08±1.47^{b}	128.42±10.68^{a}	125.00±2.19^{a}	122.20±5.34^{a}	125.63±0.51^{a}	103.71±0.39^{c}	34.33±0.81^{d}	27.11±1.02^{e}	29.16±0.54^{de}	7.05

∑SFA; sum of saturated fatty acids, ∑UFA; sum of unsaturated fatty acids.

Table S7. Fatty acid content of raw soybean flour packaged in polypropylene film and stored under refrigerated conditions ($mg·g^{-1}$, dry basis, p < 0.05, n = 3).

		Storage duration									
		Initial	1W	2W	4W	8W	12W	24W	36W	48W	LSD
	C6:0	0.02 ± 0.00^{ab}	0.03 ± 0.01^{ab}	0.02 ± 0.01^{ab}	0.10 ± 0.13^{a}	0.08 ± 0.11^{ab}	0.02 ± 0.00^{ab}	0.03 ± 0.00^{ab}	0.00 ± 0.00^{b}	0.00 ± 0.00^{b}	0.10
	C14:0	0.17 ± 0.02^{ab}	0.20 ± 0.03^{a}	0.17 ± 0.06^{ab}	0.13 ± 0.01^{bc}	0.12 ± 0.01^{c}	0.11 ± 0.01^{c}	0.13 ± 0.00^{c}	0.12 ± 0.00^{c}	0.14 ± 0.00^{bc}	0.04
	C16:0	16.60 ± 0.18^{a}	18.38 ± 2.58^{abc}	19.14 ± 2.48^{ab}	17.28 ± 0.33^{bc}	17.41 ± 0.08^{bc}	13.20 ± 0.28^{d}	16.57 ± 0.07^{c}	16.39 ± 0.07^{c}	20.34 ± 0.17^{a}	2.07
	C16:1	0.10 ± 0.00^{cd}	0.12 ± 0.02^{bc}	0.13 ± 0.01^{b}	0.10 ± 0.00^{cd}	0.10 ± 0.01^{cd}	0.09 ± 0.01^{d}	0.10 ± 0.00^{cd}	0.13 ± 0.02^{b}	0.15 ± 0.02^{a}	0.02
	C17:0	0.16 ± 0.01^{c}	0.18 ± 0.02^{bc}	0.18 ± 0.02^{b}	0.18 ± 0.00^{bc}	0.18 ± 0.01^{bc}	0.14 ± 0.00^{d}	0.17 ± 0.00^{bc}	0.16 ± 0.00^{c}	0.38 ± 0.02^{a}	0.02
	C18:0	5.76 ± 0.07^{bcd}	6.35 ± 0.88^{ab}	6.43 ± 0.84^{ab}	6.54 ± 0.16^{a}	6.30 ± 0.25^{abc}	4.51 ± 0.11^{e}	5.60 ± 0.03^{cd}	5.49 ± 0.04^{d}	6.12 ± 0.06^{abcd}	0.72
	C18:1c&t	35.96 ± 0.41^{c}	41.74 ± 6.01^{ab}	42.78 ± 5.54^{a}	37.44 ± 0.75^{bc}	38.30 ± 0.23^{abc}	28.07 ± 0.55^{d}	36.64 ± 0.20^{c}	36.85 ± 0.23^{c}	38.80 ± 0.42^{abc}	4.72
	C18:2c&t	64.36 ± 0.94^{de}	81.10 ± 12.18^{ab}	82.26 ± 10.33^{a}	72.40 ± 1.26^{bcd}	74.86 ± 0.52^{abc}	59.03 ± 1.10^{e}	69.29 ± 0.48^{cd}	71.16 ± 0.47^{cd}	75.15 ± 0.72^{cd}	9.22
	C18:3n3	12.07 ± 0.19^{ab}	14.21 ± 2.21^{a}	13.91 ± 1.69^{a}	12.41 ± 0.23^{ab}	12.99 ± 0.08^{ab}	10.66 ± 0.19^{ab}	12.66 ± 0.12^{ab}	12.79 ± 0.09^{ab}	8.94 ± 7.74^{b}	4.71
Refrigeration (4℃)	C20:0	0.63 ± 0.01^{bc}	0.68 ± 0.09^{ab}	0.74 ± 0.10^{a}	0.64 ± 0.02^{bc}	0.65 ± 0.01^{bc}	0.45 ± 0.01^{d}	0.58 ± 0.01^{c}	0.65 ± 0.04^{bc}	0.61 ± 0.02^{bc}	0.08
	C20:1n9	0.52 ± 0.04^{ab}	0.54 ± 0.05^{a}	0.54 ± 0.11^{a}	0.53 ± 0.01^{ab}	0.46 ± 0.00^{b}	0.32 ± 0.01^{c}	0.49 ± 0.02^{ab}	0.37 ± 0.01^{c}	0.33 ± 0.00^{c}	0.07
	C20:2	0.06 ± 0.01^{cd}	0.08 ± 0.01^{ab}	0.08 ± 0.02^{a}	0.06 ± 0.00^{bcd}	0.06 ± 0.00^{bcd}	0.05 ± 0.00^{d}	0.07 ± 0.00^{abc}	0.06 ± 0.00^{abcd}	0.06 ± 0.01^{abcd}	0.01
	C20:3n6&21:0	0.08 ± 0.01^{c}	0.10 ± 0.01^{a}	0.10 ± 0.02^{a}	0.08 ± 0.00^{bc}	0.08 ± 0.01^{c}	0.06 ± 0.01^{d}	0.08 ± 0.00^{c}	0.08 ± 0.00^{bc}	0.10 ± 0.00^{ab}	0.01
	C22:0	1.02 ± 0.02^{bc}	1.11 ± 0.15^{ab}	1.19 ± 0.16^{a}	0.99 ± 0.03^{bc}	1.01 ± 0.01^{bc}	0.71 ± 0.02^{d}	0.98 ± 0.01^{c}	1.01 ± 0.02^{bc}	1.11 ± 0.02^{abc}	0.13
	C22:1n9	0.03 ± 0.01^{d}	0.05 ± 0.00^{bc}	0.06 ± 0.01^{a}	0.06 ± 0.01^{ab}	0.06 ± 0.00^{a}	0.06 ± 0.01^{a}	0.04 ± 0.00^{c}	0.00 ± 0.00^{e}	0.02 ± 0.01^{d}	0.01
	C23:0	0.13 ± 0.01^{abc}	0.15 ± 0.02^{ab}	0.14 ± 0.02^{abc}	0.13 ± 0.01^{abc}	0.15 ± 0.02^{a}	0.16 ± 0.03^{a}	0.12 ± 0.00^{c}	0.12 ± 0.00^{bc}	0.14 ± 0.00^{abc}	0.03
	C24:0	0.29 ± 0.01^{c}	0.34 ± 0.04^{ab}	0.35 ± 0.05^{a}	0.30 ± 0.00^{c}	0.30 ± 0.02^{bc}	0.23 ± 0.00^{d}	0.38 ± 0.00^{a}	0.30 ± 0.02^{c}	0.28 ± 0.01^{c}	0.04
	Total	137.98 ± 1.75^{b}	165.35 ± 24.30^{a}	168.22 ± 21.43^{a}	149.36 ± 2.68^{ab}	153.13 ± 0.74^{ab}	117.86 ± 2.25^{c}	143.91 ± 0.86^{b}	145.71 ± 0.89^{b}	152.67 ± 6.71^{ab}	19..07
	∑SFA	24.88 ± 0.24^{bc}	27.52 ± 3.82^{ab}	28.46 ± 3.75^{a}	26.37 ± 0.44^{abc}	26.29 ± 0.35^{abc}	19.59 ± 0.41^{d}	24.63 ± 0.12^{bc}	24.34 ± 0.11^{c}	29.21 ± 0.23^{a}	3.09
	∑UFA	113.10 ± 1.51^{cd}	137.83 ± 20.48^{ab}	139.76 ± 17.68^{a}	122.99 ± 2.25^{bc}	126.84 ± 0.82^{abc}	98.27 ± 1.85^{d}	119.29 ± 0.77^{c}	121.36 ± 0.79^{c}	123.45 ± 6.83^{bc}	16.086

∑SFA; sum of saturated fatty acids, ∑UFA; sum of unsaturated fatty acids.

Table S8. Fatty acid content of raw soybean flour packaged in polypropylene film and stored under room temperature conditions (mg·g^{-1}, dry basis, $p < 0.05$, n = 3).

		Storage duration									
		Initial	1W	2W	4W	8W	12W	24W	36W	48W	LSD
	C6:0	0.02±0.00^{ab}	0.02±0.00^{c}	0.02±0.00^{abc}	0.02±0.00^{abc}	0.02±0.00^{bc}	0.02±0.00^{d}	0.02±0.00^{a}	0.00±0.00^{e}	0.00±0.00^{e}	0.00
	C14:0	0.17±0.02^{b}	0.19±0.01^{ab}	0.20±0.03^{a}	0.13±0.01^{c}	0.12±0.01^{cd}	0.10±0.01^{d}	0.12±0.00^{cd}	0.13±0.00^{c}	0.14±0.00^{c}	0.02
	C16:0	16.60±0.18^{bc}	16.67±0.08^{bc}	18.90±1.63^{a}	16.95±0.48^{b}	16.89±0.12^{bc}	12.60±0.12^{d}	16.12±0.06^{bc}	15.92±0.09^{c}	19.60±0.16^{a}	0.99
	C16:1	0.10±0.00^{d}	0.11±0.00^{cd}	0.12±0.02^{bc}	0.11±0.01^{cd}	0.11±0.01^{bcd}	0.08±0.00^{e}	0.10±0.00^{d}	0.13±0.01^{b}	0.15±0.01^{a}	0.01
	C17:0	0.16±0.01^{cde}	0.17±0.00^{cde}	0.18±0.01^{b}	0.17±0.01^{bc}	0.17±0.00^{bcd}	0.14±0.00^{f}	0.16±0.00^{de}	0.16±0.00^{e}	0.36±0.01^{a}	0.01
	C18:0	5.76±0.07^{bc}	5.76±0.03^{bc}	6.31±0.55^{a}	6.02±0.35^{ab}	5.85±0.04^{b}	4.29±0.06^{e}	5.45±0.01^{cd}	5.31±0.05^{d}	5.91±0.05^{b}	0.38
	C18:1c&t	35.96±0.41^{bc}	37.73±0.11^{ab}	42.16±3.61^{a}	37.12±1.19^{abc}	32.05±8.93^{c}	26.40±0.26^{d}	35.59±0.15^{bc}	35.46±0.29^{bc}	38.02±0.46^{ab}	5.57
	C18:2c&t	64.36±0.94^{d}	72.83±0.34^{b}	80.54±6.73^{a}	71.89±2.50^{bc}	72.97±0.79^{b}	55.66±0.47^{e}	67.41±0.46^{d}	67.94±0.47^{cd}	73.15±1.07b	4.24
	C18:3n3	12.07±0.19^{c}	12.69±0.08^{bc}	13.58±1.10^{a}	12.30±0.47^{bc}	12.60±0.17^{bc}	9.94±0.12^{d}	12.26±0.12^{bc}	12.03±0.12^{c}	12.85±0.25^{b}	0.73
	C20:0	0.63±0.01^{b}	0.63±0.01^{b}	0.71±0.06^{a}	0.63±0.01^{b}	0.63±0.01^{b}	0.43±0.01^{d}	0.57±0.00^{c}	0.69±0.01^{a}	0.62±0.01^{b}	0.04
Room temperature (20℃)	C20:1n9	0.52±0.04^{ab}	0.51±0.01^{abc}	0.55±0.07^{a}	0.51±0.03^{abc}	0.46±0.02^{c}	0.31±0.02^{e}	0.47±0.01^{a}	0.36±0.00^{de}	0.38±0.04^{d}	0.05
	C20:2	0.06±0.01^{ab}	0.07±0.01^{a}	0.07±0.02^{a}	0.06±0.01^{ab}	0.07±0.00^{a}	0.05±0.01^{b}	0.07±0.00^{ab}	0.06±0.00^{ab}	0.07±0.00^{a}	0.01
	C20:3n6&21:0	0.08±0.01^{c}	0.08±0.00^{bc}	0.10±0.00^{a}	0.08±0.01^{c}	0.08±0.01^{c}	0.06±0.01^{d}	0.08±0.00^{c}	0.08±0.00^{c}	0.09±0.01^{ab}	0.01
	C22:0	1.02±0.02^{b}	1.02±0.00^{b}	1.14±0.10^{a}	0.99±0.03^{bc}	0.99±0.02^{bc}	0.69±0.01^{d}	0.94±0.00^{c}	0.95±0.00^{c}	1.10±0.02^{a}	0.06
	C22:1n9	0.03±0.01^{c}	0.05±0.00^{b}	0.05±0.01^{b}	0.05±0.01^{b}	0.07±0.01^{a}	0.05±0.01^{b}	0.06±0.00^{b}	0.00±0.00^{d}	0.01±0.01^{d}	0.01
	C23:0	0.13±0.01$_{abc}$	0.14±0.00^{ab}	0.14±0.02^{a}	0.12±0.02^{cd}	0.12±0.01^{bcd}	0.12±0.01^{cd}	0.12±0.00^{cd}	0.11±0.01^{d}	0.13±0.01^{abcd}	0.02
	C24:0	0.29±0.01^{bc}	0.30±0.01^{b}	0.34±0.03^{a}	0.28±0.01^{bcd}	0.29±0.01^{bc}	0.21±0.02^{e}	0.36±0.00^{a}	0.27±0.01^{cd}	0.26±0.01^{d}	0.02
	Total	137.98±1.75^{d}	148.97±0.51^{bc}	165.13±13.92^{a}	147.45±4.71^{bcd}	143.51±8.19^{bcd}	111.15±1.01^{e}	139.89±0.79^{cd}	139.59±0.97^{cd}	152.83±1.99^{b}	9.78
	∑SFA	24.88±0.24^{bc}	24.99±0.11^{bc}	28.06±2.44^{a}	25.41±0.78^{b}	25.18±0.17^{b}	18.66±0.17^{d}	23.93±0.07^{bc}	23.63±0.16^{c}	28.21±0.23^{a}	1.49
	∑UFA	113.10±1.51^{d}	123.99±0.44^{bc}	137.07±11.49^{a}	122.04±4.15^{bc}	118.33±8.31^{bcd}	92.49±0.84^{e}	115.95±0.72^{cd}	115.96±0.82^{cd}	124.62±1.76^{b}	8.59

∑SFA; sum of saturated fatty acids, ∑UFA; sum of unsaturated fatty acids.

Table S9. Fatty acid content of raw soybean flour packaged in polypropylene film and stored under high temperature conditions (mg·g^{-1}, dry basis, $p < 0.05$, n = 3).

		Storage duration									
		Initial	1W	2W	4W	8W	12W	24W	36W	48W	LSD
	C6:0	0.02±0.00^{b}	0.02±0.00^{b}	0.02±0.00^{b}	0.02±0.00^{b}	0.02±0.00^{b}	0.02±0.00^{c}	0.04±0.01^{a}	0.00±0.00^{d}	0.00±0.00d	0.00
	C14:0	0.17±0.02^{b}	0.18±0.00^{ab}	0.20±0.01^{a}	0.12±0.00^{cd}	0.13±0.01^{cd}	0.11±0.01$_{d}$	0.13±0.01^{cd}	0.13±0.01^{c}	0.14±0.00^{c}	0.02
	C16:0	16.60±0.18^{c}	16.91±0.20^{c}	18.92±0.32^{b}	16.63±1.33^{c}	16.89±0.54^{c}	13.59±0.17^{d}	17.00±0.19^{c}	16.58±0.22^{c}	20.24±0.31a	0.89
	C16:1	0.10±0.00^{cd}	0.10±0.00^{bc}	0.12±0.01^{b}	0.10±0.01^{cd}	0.10±0.01^{c}	0.09±0.00^{d}	0.11±0.02^{bc}	0.11±0.01^{bc}	0.15±0.01^{a}	0.02
	C17:0	0.16±0.01^{bc}	0.18±0.01^{b}	0.17±0.01^{b}	0.17±0.02^{bc}	0.17±0.00^{b}	0.15±0.01^{c}	0.16±0.02^{bc}	0.17±0.00^{bc}	0.38±0.01^{a}	0.02
	C18:0	5.76±0.07^{d}	5.85±0.06^{cd}	6.32±0.10^{ab}	5.75±0.44^{d}	6.47±0.17^{a}	4.68±0.13^{e}	5.74±0.12^{d}	5.56±0.07^{d}	6.12±0.12^{bc}	0.31
	C18:1c&t	35.96±0.41^{d}	38.33±0.46^{bc}	42.17±0.72^{a}	36.56±2.98^{cd}	36.82±1.18^{bcd}	28.78±0.40^{e}	36.61±1.88^{cd}	36.92±0.50^{bcd}	38.89±0.66^{d}	2.26
	C18:2c&t	64.36±0.94^{de}	74.03±0.82^{b}	80.62±1.24^{a}	70.54±5.66^{bc}	71.69±2.35^{b}	60.29±0.79^{e}	64.70±7.77^{cde}	69.74±1.17^{bcd}	72.77±1.69^{b}	5.88
	C18:3n3	12.07±0.19^{bc}	12.93±0.14^{ab}	13.54±0.22^{a}	12.04±0.96^{bc}	12.28±0.41^{b}	10.70±0.17^{d}	11.10±1.69^{cd}	11.85±0.19^{bcd}	11.98±0.29^{bc}	1.17
High temperature (45℃)	C20:0	0.63±0.01^{bc}	0.63±0.01^{b}	0.72±0.02^{b}	0.61±0.05^{bc}	0.63±0.01^{b}	0.47±0.00^{d}	0.59±0.02^{c}	0.71±0.00^{a}	0.65±0.02^{b}	0.04
	C20:1n9	0.52±0.04^{ab}	0.52±0.01^{ab}	0.57±0.02^{a}	0.52±0.05^{ab}	0.46±0.03^{b}	0.32±0.02^{c}	0.55±0.04^{a}	0.38±0.01^{c}	0.38±0.08^{c}	0.07
	C20:2	0.06±0.01^{cd}	0.07±0.01^{ab}	0.08±0.01^{a}	0.06±0.01^{cd}	0.06±0.01^{cd}	0.05±0.00^{d}	0.06±0.01^{d}	0.07±0.01^{bcd}	0.07±0.01^{abc}	0.01
	C20:3n6&21:0	0.08±0.01^{bc}	0.09±0.00^{ab}	0.09±0.01^{ab}	0.09±0.01^{a}	0.08±0.01^{ab}	0.06±0.01^{c}	0.08±0.00^{ab}	0.09±0.01^{ab}	0.09±0.00^{a}	0.01
	C22:0	1.02±0.02^{bc}	1.04±0.02^{b}	1.15±0.01^{a}	0.98±0.08^{c}	0.99±0.02^{bc}	0.73±0.02^{d}	0.98±0.03^{bc}	1.02±0.01^{bc}	1.16±0.04^{a}	0.06
	C22:1n9	0.03±0.01^{g}	0.05±0.00^{ef}	0.06±0.01^{de}	0.05±0.01^{f}	0.08±0.00^{c}	0.09±0.01^{b}	0.06±0.01^{d}	0.16±0.01^{a}	0.04±0.01^{f}	0.01
	C23:0	0.13±0.01^{abc}	0.13±0.00^{abc}	0.14±0.01^{ab}	0.12±0.02^{c}	0.14±0.01^{a}	0.13±0.02^{bc}	0.12±0.00^{c}	0.12±0.00^{bc}	0.14±0.01^{ab}	0.02
	C24:0	0.29±0.01^{c}	0.33±0.01^{b}	0.35±0.01^{b}	0.28±0.03^{c}	0.29±0.02^{c}	0.24±0.01^{d}	0.38±0.00^{a}	0.29±0.01^{c}	0.28±0.01^{c}	0.02
	Total	137.98±1.75^{c}	151.39±1.68^{b}	165.24±2.63^{a}	144.64±11.63^{bc}	147.31±4.72^{bc}	120.50±1.64^{d}	138.41±11.58^{c}	143.90±2.19^{bc}	153.47±3.22^{b}	10.27
	∑SFA	24.88±0.24^{b}	25.36±0.27^{b}	28.08±0.45^{a}	24.77±1.97^{b}	25.83±0.76^{b}	20.18±0.30^{c}	25.23±0.32^{b}	24.66±0.31^{b}	29.19±0.50^{a}	1.32
	∑UFA	113.10±1.51^{c}	126.04±1.41^{b}	137.16±2.18^{a}	119.87±9.67^{bc}	121.49±3.96^{bc}	100.32±1.39^{d}	113.18±11.30^{c}	119.24±1.88^{bc}	124.28±2.72^{b}	9.20

∑SFA; sum of saturated fatty acids, ∑UFA; sum of unsaturated fatty acids.

Table S10. Fatty acid content of roasted soybean flour packaged in polypropylene film and stored under refrigerated conditions (mg·g^{-1}, dry basis, $p < 0.05$, n = 3).

		Storage duration								
		Initial	1W	2W	4W	8W	12W	24W	36W	48W	LSD
	C6:0	0.04±0.00^{b}	0.04±0.00^{a}	0.04±0.00^{b}	0.03±0.00^{c}	0.03±0.00^{b}	0.02±0.00^{d}	0.03±0.00^{b}	0.00±0.00^{e}	0.00±0.00^{e}	0.00
	C14:0	0.19±0.02^{a}	0.19±0.01^{a}	0.19±0.01^{a}	0.11±0.00^{cd}	0.12±0.00^{bc}	0.10±0.00^{d}	0.13±0.00^{b}	0.12±0.00^{bc}	0.13±0.00^{b}	0.02
	C16:0	16.36±0.21^{cde}	16.97±0.27^{bcd}	18.22±1.26^{b}	15.59±0.57^{de}	17.33±0.09^{bc}	13.74±0.17^{f}	16.77±0.08^{bcd}	14.89±2.61^{ef}	20.32±0.17^{a}	1.70
	C16:1	0.10±0.00^{cd}	0.11±0.01^{bc}	0.13±0.02^{a}	0.09±0.01^{cd}	0.11±0.01^{bc}	0.09±0.01^{d}	0.10±0.00^{cd}	0.12±0.01^{ab}	0.13±0.01^{a}	0.02
	C17:0	0.16±0.01^{cde}	0.17±0.00^{bcd}	0.18±0.01^{b}	0.16±0.01^{de}	0.18±0.01^{bc}	0.15±0.00^{e}	0.17±0.00^{bcde}	0.17±0.00^{cde}	0.36±0.02^{a}	0.02
	C18:0	5.63±0.07^{c}	5.81±0.10^{bc}	6.09±0.42^{ab}	5.48±0.20^{c}	6.27±0.29^{a}	4.63±0.06^{d}	5.64±0.04^{c}	5.48±0.06^{c}	6.08±0.07^{ab}	0.33
	C18:1c&t	35.33±0.47^{de}	38.05±0.53^{bc}	40.30±2.70^{a}	34.01±1.28^{e}	37.76±0.42^{bc}	29.28±0.79^{f}	36.86±0.18^{cd}	36.62±0.34^{cd}	39.08±0.33^{ab}	1.85
	C18:2c&t	63.13±0.83^{de}	73.30±0.87^{a}	76.67±4.99^{a}	65.97±2.46^{cd}	73.88±0.72^{a}	61.52±1.29^{e}	69.32±0.33^{bc}	69.77±0.72^{b}	73.71±1.33^{a}	3.48
	C18:3n3	11.89±0.16^{bc}	12.75±0.15^{a}	12.89±0.85^{a}	11.35±0.40^{cd}	12.82±0.13^{a}	11.10±0.28^{d}	12.61±0.07^{a}	12.36±0.15^{ab}	12.87±0.29^{a}	0.61
Refrigeration	C20:0	0.61±0.00^{bc}	0.62±0.01^{abc}	0.70±0.05^{a}	0.59±0.02^{c}	0.65±0.01^{abc}	0.46±0.02^{d}	0.59±0.00^{c}	0.68±0.01^{ab}	0.68±0.14^{ab}	0.09
(4℃)	C20:1n9	0.51±0.03^{abc}	0.53±0.04^{ab}	0.57±0.06^{a}	0.47±0.04^{c}	0.47±0.01^{c}	0.35±0.03^{d}	0.49±0.02^{bc}	0.38±0.00^{d}	0.37±0.06^{d}	0.06
	C20:2	0.05±0.01^{d}	0.06±0.01^{abc}	0.06±0.01^{abc}	0.05±0.00^{cd}	0.07±0.00^{a}	0.06±0.01^{bcd}	0.06±0.00^{abc}	0.06±0.00^{abc}	0.07±0.01^{ab}	0.01
	C20:3n6&21:0	0.08±0.01^{bcd}	0.09±0.01^{a}	0.09±0.00^{a}	0.07±0.01^{cd}	0.08±0.01^{ab}	0.06±0.01^{d}	0.08±0.00^{abc}	0.09±0.00^{ab}	0.09±0.01^{ab}	0.01
	C22:0	0.99±0.01^{b}	1.02±0.03^{b}	1.11±0.08^{a}	0.90±0.03^{c}	1.00±0.01^{b}	0.75±0.03^{d}	0.98±0.01^{b}	0.99±0.01^{b}	1.10±0.02^{a}	0.05
	C22:1n9	0.05±0.01^{bc}	0.05±0.01^{c}	0.07±0.02^{a}	0.03±0.00^{d}	0.06±0.01^{ab}	0.05±0.01^{bc}	0.04±0.01^{cd}	0.00±0.00^{e}	0.00±0.00^{e}	0.02
	C23:0	0.12±0.00^{bcd}	0.13±0.01^{abcd}	0.15±0.01^{ab}	0.12±0.01^{cd}	0.14±0.02^{abc}	0.12±0.03^{d}	0.12±0.00^{cd}	0.13±0.01^{bcd}	0.15±0.01^{a}	0.02
	C24:0	0.29±0.01^{c}	0.33±0.00^{b}	0.34±0.02^{b}	0.25±0.01^{d}	0.29±0.01^{c}	0.24±0.01^{e}	0.36±0.00^{a}	0.28±0.01^{c}	0.28±0.01^{c}	0.02
	Total	135.56±1.77^{e}	150.23±1.91^{bc}	157.81±10.41^{a}	135.27±4.90^{e}	151.26±1.23^{abc}	122.72±2.60^{f}	144.37±0.69^{cd}	142.14±2.60^{de}	155.42±1.97^{ab}	7.20
	∑SFA	24.48±0.30^{cde}	25.38±0.40^{bc}	27.10±1.85^{b}	23.31±0.75^{de}	26.09±0.34^{bc}	20.27±0.25^{f}	24.87±0.13^{cd}	22.82±2.56^{e}	29.19±0.28^{a}	1.90
	∑UFA	111.08±1.47^{c}	124.85±1.53^{ab}	130.71±8.57^{a}	111.96±4.18^{c}	125.17±1.25^{ab}	102.45±2.37^{d}	119.50±0.57^{b}	119.32±1.20^{b}	126.23±1.88^{a}	5.94

∑SFA; sum of saturated fatty acids, ∑UFA; sum of unsaturated fatty acids.

Table S11. Fatty acid content of roasted soybean flour packaged in polypropylene film and stored under room temperature conditions ($mg \cdot g^{-1}$, dry basis, $p < 0.05$, n = 3).

		Storage duration								
		Initial	1W	2W	4W	8W	12W	24W	36W	48W	LSD
Room temperature (20℃)	C6:0	0.04 ± 0.00^{bc}	0.04 ± 0.00^{a}	0.04 ± 0.00^{b}	0.03 ± 0.00^{d}	0.03 ± 0.00^{c}	0.02 ± 0.00^{e}	0.03 ± 0.00^{d}	0.00 ± 0.00^{f}	0.00 ± 0.00^{f}	0.00
	C14:0	0.19 ± 0.02^{a}	0.19 ± 0.00^{a}	0.21 ± 0.02^{a}	0.12 ± 0.01^{b}	0.12 ± 0.00^{b}	0.11 ± 0.00^{b}	0.13 ± 0.00^{b}	0.13 ± 0.01^{b}	0.12 ± 0.00^{b}	0.02
	C16:0	16.36 ± 0.21^{bc}	16.96 ± 0.12^{b}	19.93 ± 0.46^{a}	15.68 ± 0.34^{c}	17.00 ± 0.05^{b}	13.16 ± 0.15^{d}	16.44 ± 0.08^{bc}	16.57 ± 1.23^{b}	17.09 ± 0.19^{b}	0.80
	C16:1	0.10 ± 0.00^{cd}	0.10 ± 0.02^{cd}	0.12 ± 0.01^{ab}	0.10 ± 0.01^{cd}	0.11 ± 0.01^{bc}	0.08 ± 0.00^{d}	0.12 ± 0.03^{abc}	0.14 ± 0.01^{a}	0.14 ± 0.01^{a}	0.02
	C17:0	0.16 ± 0.01^{cd}	0.17 ± 0.01^{c}	0.19 ± 0.01^{b}	0.17 ± 0.00^{c}	0.17 ± 0.01^{c}	0.15 ± 0.00^{d}	0.16 ± 0.00^{cd}	0.17 ± 0.01^{c}	0.29 ± 0.01^{a}	0.01
	C18:0	5.63 ± 0.07^{c}	5.80 ± 0.05^{bc}	6.62 ± 0.15^{a}	5.79 ± 0.11^{bc}	6.03 ± 0.23^{b}	4.41 ± 0.12^{e}	5.52 ± 0.02^{c}	5.54 ± 0.40^{c}	5.13 ± 0.04^{d}	0.30
	C18:1c&t	35.33 ± 0.47^{de}	38.01 ± 0.24^{b}	44.17 ± 1.09^{a}	33.89 ± 0.80^{ef}	37.16 ± 0.30^{bc}	27.87 ± 0.44^{g}	36.04 ± 0.06^{cd}	36.91 ± 2.69^{bcd}	32.70 ± 0.33^{f}	1.78
	C18:2c&t	63.13 ± 0.83^{e}	73.22 ± 0.82^{b}	84.67 ± 2.27^{a}	65.54 ± 1.70^{de}	71.96 ± 0.78^{b}	58.63 ± 0.91^{f}	68.07 ± 0.16^{cd}	70.54 ± 5.24^{bc}	62.08 ± 0.65^{ef}	3.56
	C18:3n3	11.89 ± 0.16^{c}	12.72 ± 0.21^{b}	14.29 ± 0.40^{a}	11.24 ± 0.31^{d}	12.39 ± 0.19^{bc}	10.63 ± 0.16^{d}	12.43 ± 0.09^{bc}	12.58 ± 0.95^{b}	10.94 ± 0.11^{d}	0.66
	C20:0	0.61 ± 0.00^{cde}	0.63 ± 0.01^{cd}	0.77 ± 0.02^{a}	0.59 ± 0.02^{de}	0.63 ± 0.01^{c}	0.43 ± 0.01^{g}	0.57 ± 0.00^{e}	0.70 ± 0.06^{b}	0.52 ± 0.02^{f}	0.04
	C20:1n9	0.51 ± 0.03^{b}	0.55 ± 0.02^{a}	0.59 ± 0.01^{a}	0.48 ± 0.01^{c}	0.47 ± 0.02^{c}	0.33 ± 0.02^{e}	0.47 ± 0.01^{c}	0.37 ± 0.04^{d}	0.26 ± 0.01^{f}	0.04
	C20:2	0.05 ± 0.01^{d}	0.08 ± 0.00^{a}	0.07 ± 0.01^{ab}	0.06 ± 0.00^{cd}	0.06 ± 0.01^{bc}	0.05 ± 0.00^{cd}	0.06 ± 0.00^{bc}	0.06 ± 0.00^{bc}	0.06 ± 0.00^{cd}	0.01
	C20:3n6&21:0	0.08 ± 0.01^{bcd}	0.09 ± 0.01^{b}	0.10 ± 0.00^{a}	0.07 ± 0.01^{d}	0.08 ± 0.01^{bc}	0.07 ± 0.01^{cd}	0.08 ± 0.00^{bcd}	0.09 ± 0.00^{b}	0.09 ± 0.00^{b}	0.01
	C22:0	0.99 ± 0.01^{bc}	1.04 ± 0.01^{b}	1.20 ± 0.02^{a}	0.89 ± 0.03^{e}	0.98 ± 0.00^{cd}	0.71 ± 0.01^{f}	0.95 ± 0.00^{cd}	0.99 ± 0.08^{c}	0.94 ± 0.01^{de}	0.05
	C22:1n9	0.05 ± 0.01^{abcd}	0.04 ± 0.01^{cd}	0.05 ± 0.00^{abc}	0.04 ± 0.02^{d}	0.06 ± 0.01^{a}	0.06 ± 0.01^{ab}	0.05 ± 0.00^{bcd}	0.00 ± 0.00^{e}	0.00 ± 0.00^{e}	0.01
	C23:0	0.12 ± 0.00^{b}	0.12 ± 0.01^{b}	0.15 ± 0.02^{a}	0.12 ± 0.00^{bc}	0.13 ± 0.00^{b}	0.11 ± 0.02^{c}	0.12 ± 0.00^{bc}	0.12 ± 0.01^{bc}	0.12 ± 0.00^{b}	0.02
	C24:0	0.29 ± 0.01^{c}	0.32 ± 0.01^{b}	0.35 ± 0.01^{ab}	0.26 ± 0.03^{de}	0.29 ± 0.01^{c}	0.23 ± 0.02^{f}	0.36 ± 0.00^{a}	0.27 ± 0.02^{cd}	0.24 ± 0.01^{ef}	0.03
	Total	135.56 ± 1.77^{de}	150.10 ± 1.32^{b}	173.52 ± 4.39^{a}	135.04 ± 3.33^{de}	147.69 ± 0.79^{bc}	117.03 ± 1.67^{f}	141.59 ± 0.12^{cd}	145.17 ± 10.76^{bc}	130.73 ± 1.30^{e}	7.14
	∑SFA	24.48 ± 0.30^{bc}	25.37 ± 0.17^{b}	29.55 ± 0.66^{a}	23.70 ± 0.52^{c}	25.47 ± 0.24^{b}	19.39 ± 0.24^{d}	24.35 ± 0.09^{bc}	24.57 ± 1.81^{bc}	24.54 ± 0.22^{bc}	1.18
	∑UFA	111.08 ± 1.47^{e}	124.73 ± 1.21^{b}	143.97 ± 3.73^{a}	111.34 ± 2.82^{de}	122.22 ± 0.92^{bc}	97.64 ± 1.51^{f}	117.24 ± 0.21^{cd}	120.60 ± 8.94^{bc}	106.19 ± 1.08^{e}	5.99

∑SFA; sum of saturated fatty acids, ∑UFA; sum of unsaturated fatty acids.

Table S12. Fatty acid content of roasted soybean flour packed in polypropylene film under high temperature condition during storage ($mg·g^{-1}$, dry basis, $p < 0.05$, n = 3).

		Storage duration									
		Initial	1W	2W	4W	8W	12W	24W	36W	48W	LSD
	C6:0	0.04±0.00^{b}	0.03±0.00^{b}	0.03±0.00^{bc}	0.03±0.00^{bc}	0.04±0.00^{b}	0.02±0.00^{c}	1.51±0.02^{a}	0.00±0.00^{d}	0.00±0.00^{d}	0.01
	C14:0	0.19±0.02^{c}	0.19±0.01^{c}	0.19±0.01^{c}	0.12±0.01^{d}	0.13±0.01^{d}	0.12±0.01^{d}	0.12±0.01^{d}	0.44±0.01^{a}	0.42±0.01^{b}	0.02
	C16:0	16.36±0.21^{d}	16.93±0.06^{c}	19.94±0.68^{a}	17.74±0.33^{b}	17.27±0.11^{bc}	14.85±0.11^{f}	16.04±0.09^{de}	15.80±0.10^{e}	16.14±0.11^{de}	0.47
	C16:1	0.10±0.00^{d}	0.11±0.00^{d}	0.13±0.01^{bc}	0.11±0.01^{cd}	0.11±0.01^{d}	0.10±0.00^{d}	0.17±0.03^{bc}	0.14±0.01^{b}	0.11±0.02^{d}	0.02
	C17:0	0.16±0.01^{de}	0.18±0.01^{cd}	0.19±0.00^{b}	0.19±0.01^{bc}	0.18±0.00^{c}	0.16±0.00^{e}	0.16±0.00^{e}	0.16±0.00^{e}	0.27±0.01^{a}	0.01
	C18:0	5.63±0.07^{cd}	5.82±0.03^{c}	6.65±0.22^{a}	6.48±0.40^{ab}	6.29±0.33^{b}	5.04±0.12^{fg}	5.40±0.04^{de}	5.22±0.03^{ef}	4.85±0.05^{g}	0.37
	C18:1c&t	35.33±0.47^{bcd}	37.96±0.25^{bc}	44.38±1.51^{a}	38.50±0.65^{ab}	31.46±10.27^{de}	32.04±0.46^{cde}	26.64±0.14^{ef}	24.53±0.31^{f}	21.90±0.19^{f}	5.96
	C18:2c&t	63.13±0.83^{d}	73.06±0.77^{bc}	85.16±3.18^{a}	74.24±1.49^{b}	71.67±0.30^{c}	64.76±0.72^{d}	6.05±0.11^{e}	3.86±0.17^{f}	3.30±0.14^{f}	2.16
	C18:3n3	11.89±0.16^{c}	12.69±0.19^{b}	14.41±0.58^{a}	12.69±0.28^{b}	12.11±0.04^{c}	11.22±0.14^{d}	0.45±0.01^{e}	0.27±0.01^{e}	0.24±0.02^{e}	0.40
High temperature (45℃)	C20:0	0.61±0.00^{d}	0.63±0.01^{c}	0.77±0.01^{a}	0.66±0.02^{b}	0.62±0.01^{cd}	0.51±0.00^{f}	0.57±0.00^{e}	0.61±0.01^{d}	0.58±0.00^{e}	0.02
	C20:1n9	0.51±0.03^{bc}	0.54±0.02^{bc}	0.56±0.04^{b}	0.53±0.01^{bc}	0.50±0.02^{c}	0.39±0.00^{d}	1.32±0.08^{a}	0.28±0.00^{e}	0.25±0.01^{e}	0.06
	C20:2	0.05±0.01^{d}	0.07±0.01^{ab}	0.07±0.00^{a}	0.06±0.00^{bc}	0.06±0.01^{bc}	0.06±0.01^{cd}	0.00±0.00^{e}	0.00±0.00^{e}	0.00±0.00^{e}	0.01
	C20:3n6&21:0	0.08±0.01^{de}	0.09±0.01^{cd}	0.10±0.00^{bc}	0.08±0.01^{de}	0.09±0.01^{cde}	0.07±0.01^{e}	0.07±0.00^{e}	0.12±0.00^{ab}	0.13±0.01^{a}	0.02
	C22:0	0.99±0.01^{c}	1.03±0.01^{b}	1.22±0.04^{a}	1.03±0.02^{b}	0.99±0.01^{c}	0.85±0.01^{e}	0.93±0.01^{d}	1.00±0.01^{c}	1.01±0.01^{bc}	0.03
	C22:1n9	0.05±0.01^{b}	0.05±0.01^{b}	0.06±0.01^{b}	0.04±0.00^{b}	0.06±0.01^{b}	0.09±0.00^{b}	0.10±0.01^{b}	0.27±0.01^{a}	0.22±0.12^{a}	0.07
	C23:0	0.12±0.00^{bcd}	0.13±0.01^{bc}	0.15±0.01^{a}	0.13±0.00^{bc}	0.12±0.00^{bcd}	0.11±0.01^{d}	0.11±0.01^{d}	0.13±0.01^{b}	0.12±0.01^{cd}	0.01
	C24:0	0.29±0.01^{c}	0.31±0.01^{bc}	0.36±0.01^{a}	0.30±0.01^{c}	0.30±0.01^{c}	0.24±0.01^{d}	0.38±0.01^{a}	0.31±0.01^{bc}	0.32±0.03^{b}	0.02
	Total	135.56±1.77^{cd}	149.81±1.27^{b}	174.37±6.20^{a}	152.93±3.00^{b}	141.98±10.55^{c}	130.63±1.30^{d}	60.02±0.25^{e}	53.13±0.65^{ef}	49.86±0.46^{f}	7.36
	∑SFA	24.48±0.30^{d}	25.34±0.11^{c}	29.60±0.96^{a}	26.76±0.72^{b}	26.01±0.42^{bc}	21.97±0.05^{e}	25.29±0.16^{c}	23.79±0.16^{d}	23.84±0.17^{d}	0.77
	∑UFA	111.08±1.47^{cd}	124.47±1.17^{b}	144.77±5.26^{a}	126.17±2.42^{b}	115.97±10.16^{c}	108.66±1.30^{d}	34.73±0.09^{e}	29.34±0.50^{ef}	26.02±0.30^{f}	6.82

∑SFA; sum of saturated fatty acids, ∑UFA; sum of unsaturated fatty acid.

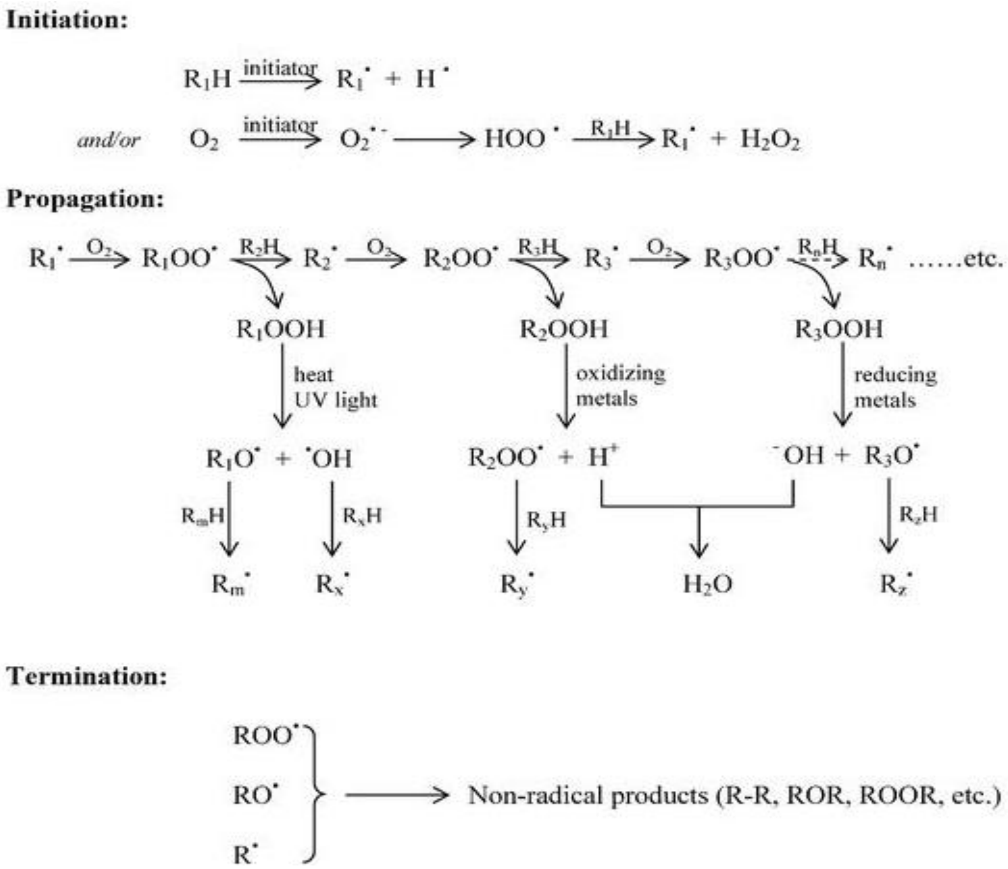

Figure S1. Three steps of lipid autoxidation and the pathways of complex chemical changes that occur through free radical chain mechanisms.

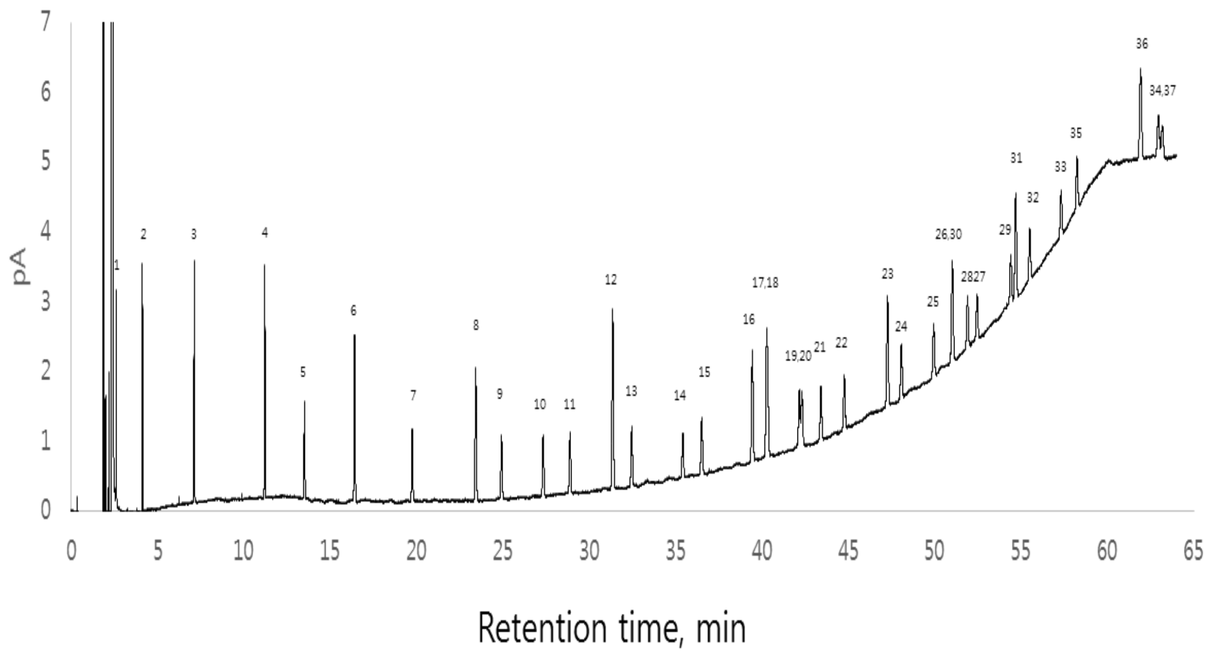

Figure S2. Chromatograms of the 37-fatty acid methyl ester standard mixture. 1, C4:0; 2, C6:0; 3, C8:0; 4, C10:0; 5, C11:0; 6, C12:0; 7, C13:0; 8, C14:0; 9, C14:1; 10, C15:0; 11, C15:1; 12, C16:0; 13, C16:1; 14, C17:0; 15, C17:1; 16, C18:0; 17 and 18, C18:1n9 t and c; 19 and 20, C18:2n6 t and c; 21, C18:3n6; 22, C18:3n3; 23, C20:0; 24, C20:1; 25, C20:2; 26 and 30, C20:3n6 and C21:0; 27, C20:3n3; 28, C20:4n6; 29, C20:5n3; 31, C22:0; 32, C22:1n9; 33, C22:2; 35, C23:0; 36, C24:0; 34 and 37, C22:6n3 and C24:1n9.

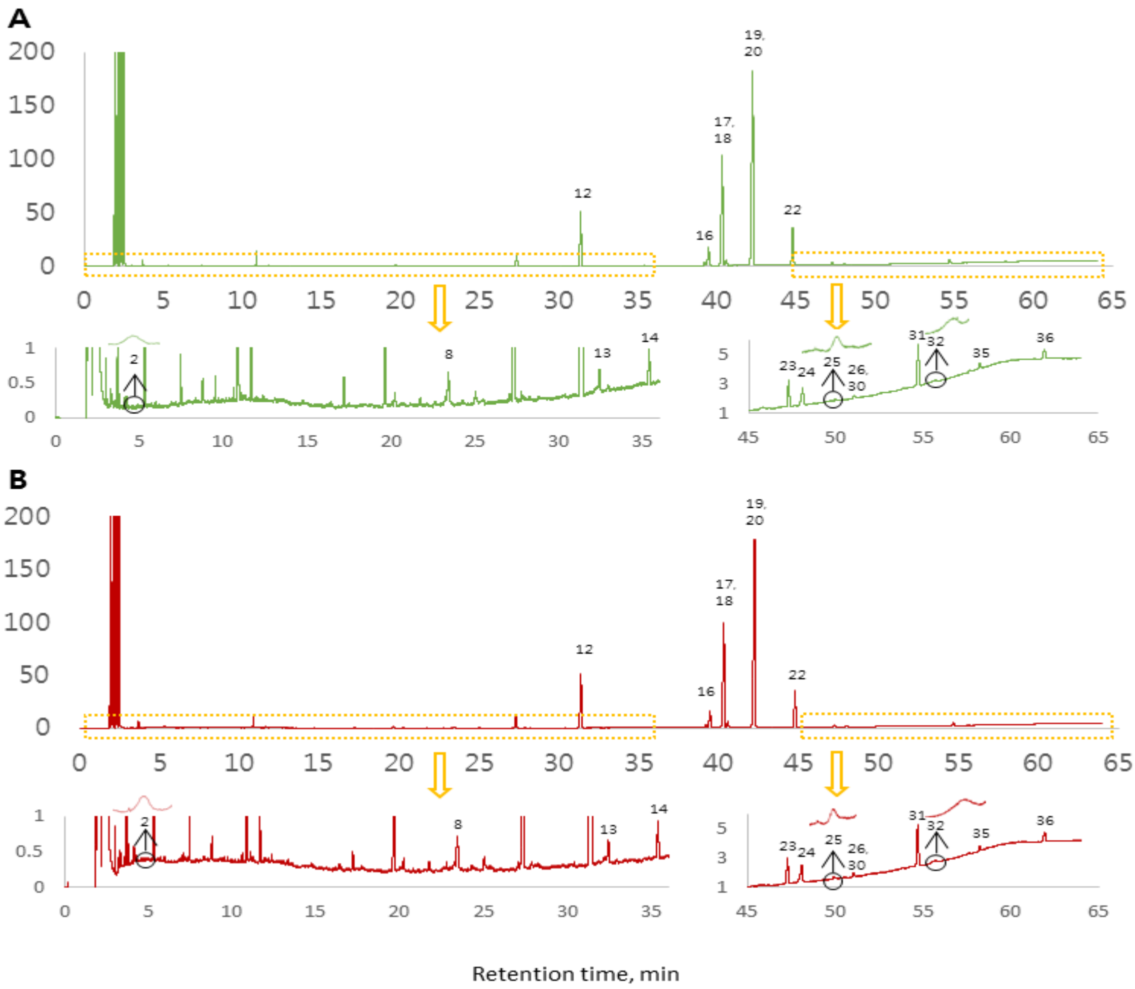

Figure S3. Chromatograms of the raw soybean flour (A) and roasted soybean flour (B) prior to storage. Arrow represents exclusive region at mentioned retention time.

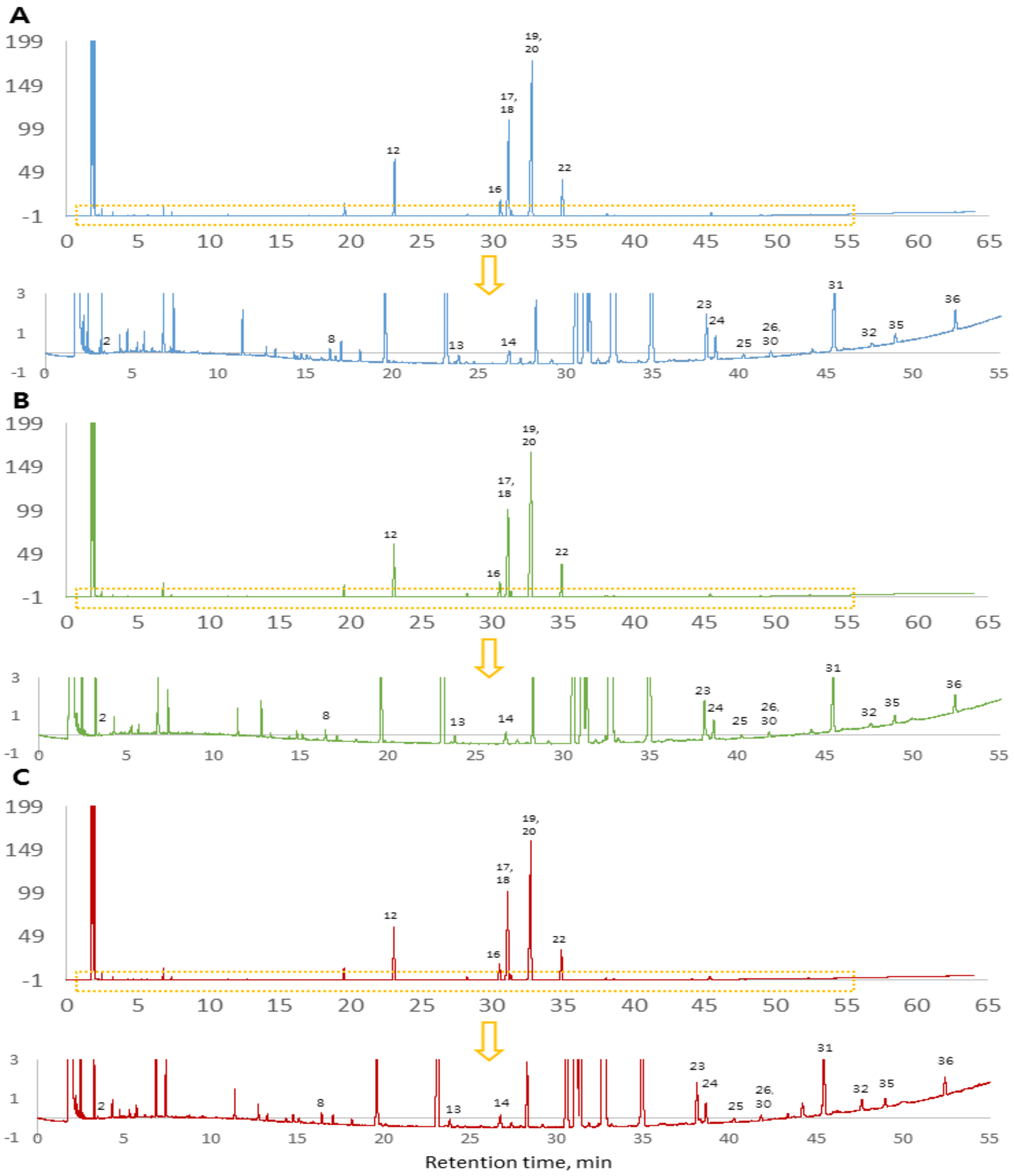

Figure S4. Chromatograms of raw soybean flour packed in polyethylene film. Stored in refrigeration (4°C) (A), room temperature (20°C) (B), and high temperature (45°C) (C) conditions for 48 weeks. Arrow represents exclusive region at mentioned retention time.

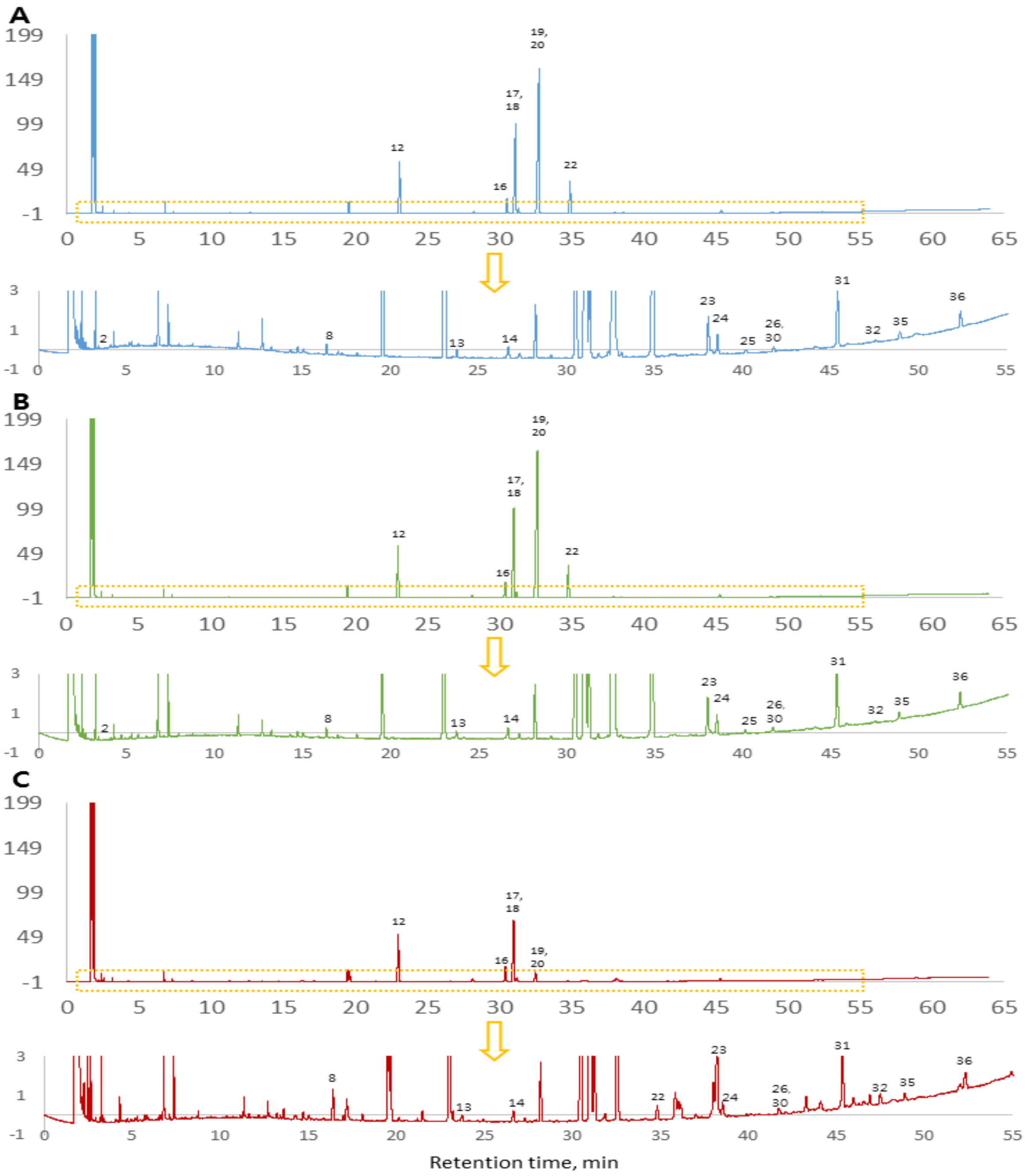

Figure S5. Chromatograms of roasted soybean flour packed in polyethylene film. Stored in refrigeration (4°C) (A), room temperature (20°C) (B), and high temperature (45°C) (C) conditions for 48 weeks. Arrow represents exclusive region at mentioned retention time.

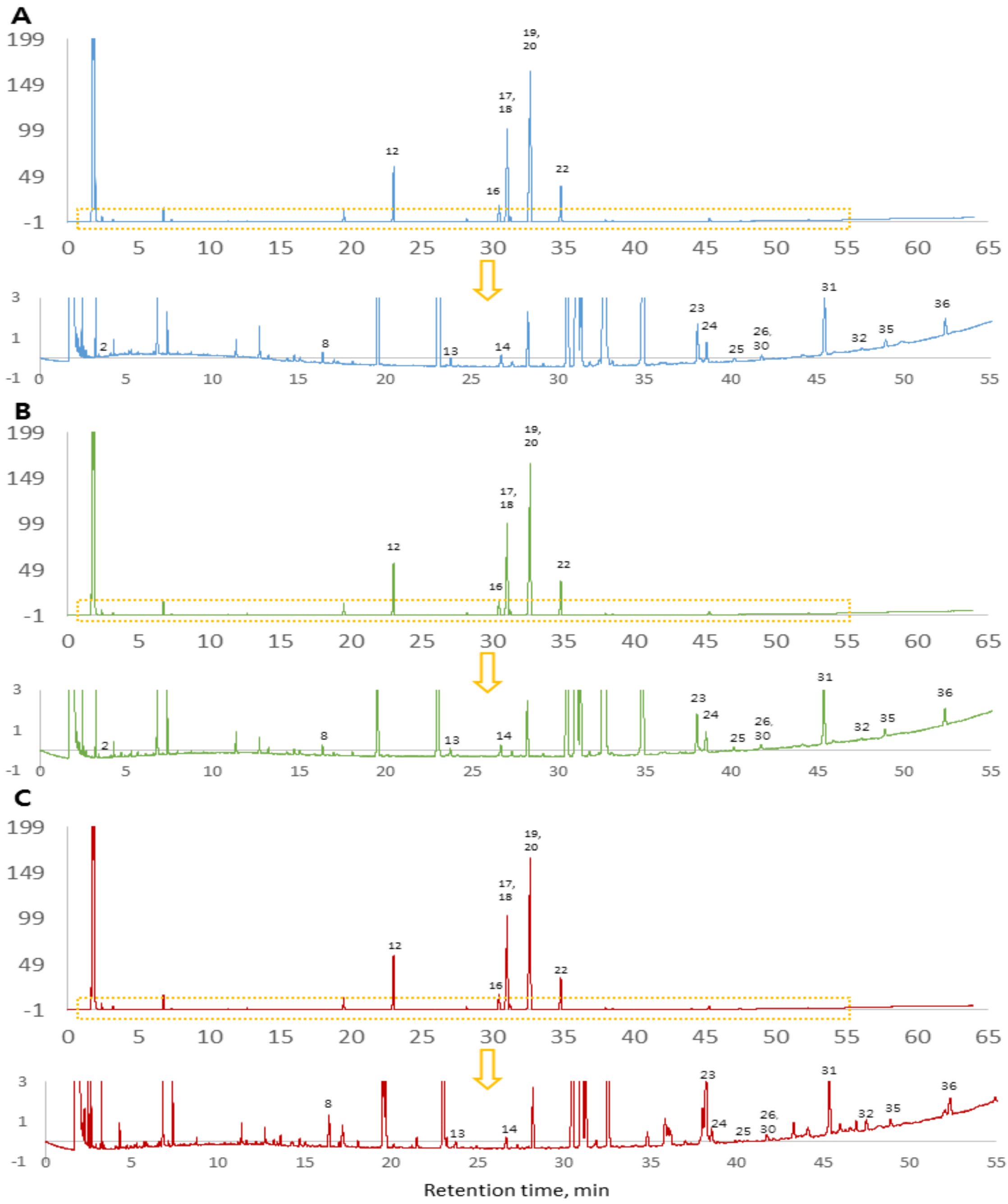

Figure S6. Chromatograms of raw soybean flour packed in polypropylene film. Stored in refrigeration (4°C) (A), room temperature (20°C) (B), and high temperature (45°C) (C) conditions for 48 weeks. Arrow represents exclusive region at mentioned retention time.

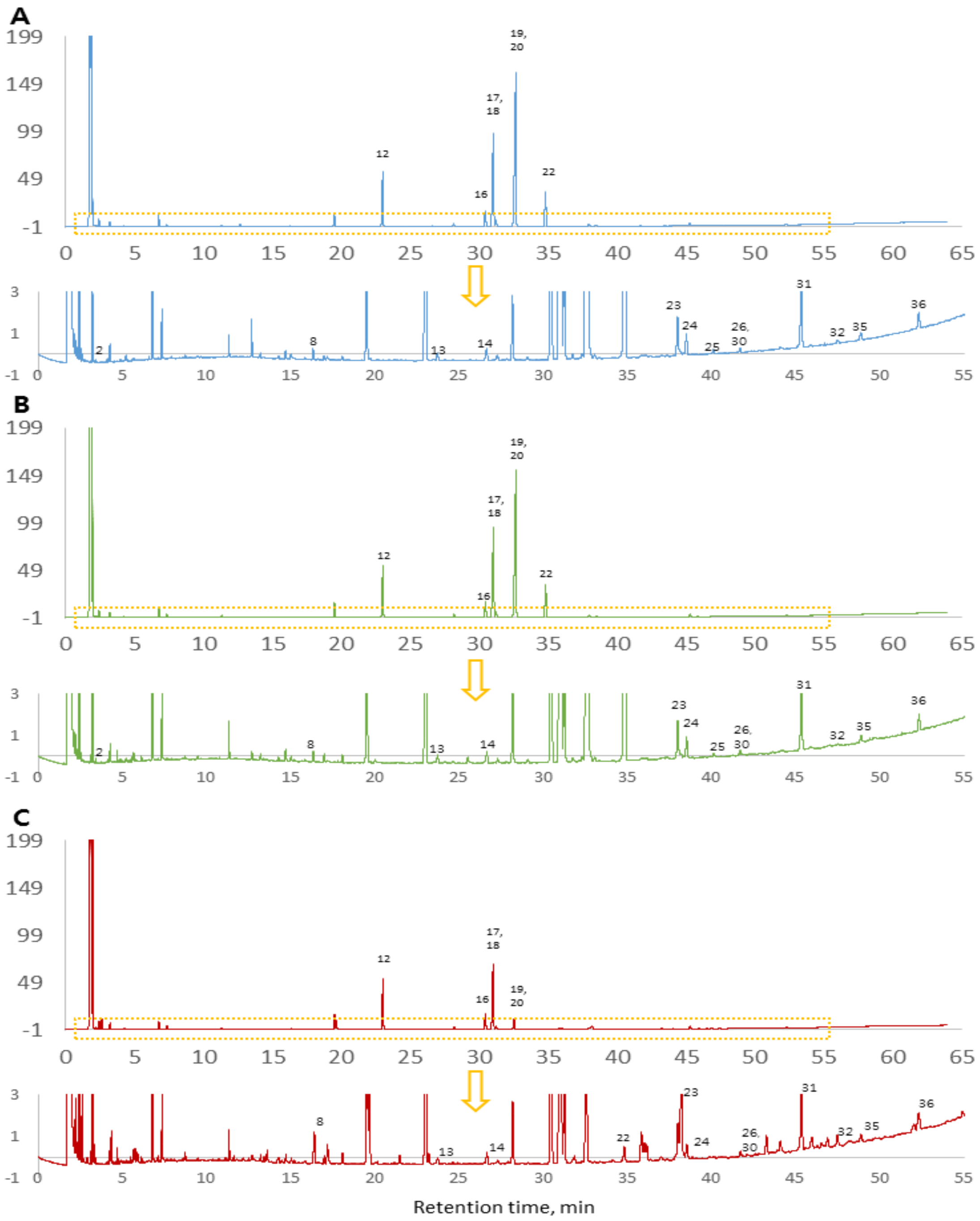

Figure S7. Chromatograms of roasted soybean flour packed in polypropylene film. Stored in refrigeration (4°C) (A), room temperature (20°C) (B), and high temperature (45°C) (C) conditions for 48 weeks. Arrow represents exclusive region at mentioned retention time.
